# Lignin-Based Porous Supraparticles for Carbon Capture

**DOI:** 10.1021/acsnano.0c10307

**Published:** 2021-03-29

**Authors:** Bin Zhao, Maryam Borghei, Tao Zou, Ling Wang, Leena-Sisko Johansson, Johanna Majoinen, Mika H. Sipponen, Monika Österberg, Bruno D. Mattos, Orlando J. Rojas

**Affiliations:** † Department of Bioproducts and Biosystems, School of Chemical Engineering, Aalto University, P.O. Box 16300, FIN-00076 Espoo, Finland; ‡ Department of Materials and Environmental Chemistry, Stockholm University, Svante Arrhenius väg 16 C, 106 91 Stockholm, Sweden; § Bioproduct Institute, Departments of Chemical & Biological Engineering, Chemistry, and Wood Science, The University of British Columbia, 2360 East Mall, Vancouver, BC V6T 1Z3, Canada

**Keywords:** lignin particles, cellulose nanofibrils, evaporation-induced self-assembly, carbon supraparticles, CO_2_ capture

## Abstract

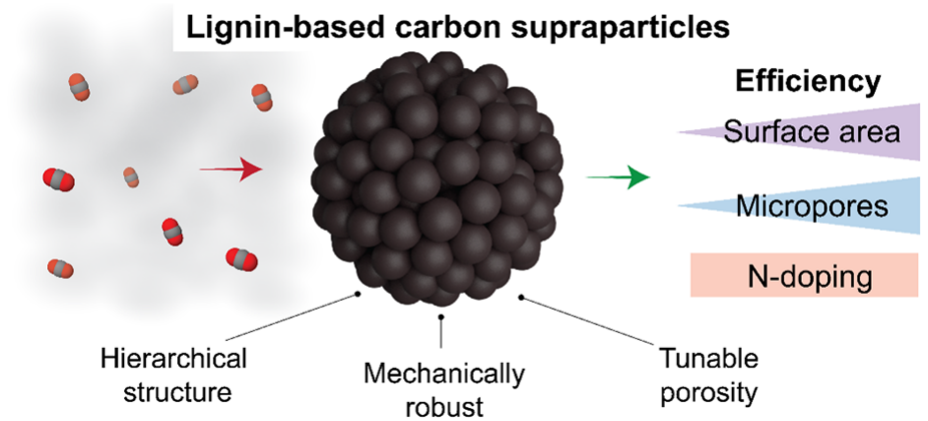

Multiscale carbon supraparticles (SPs) are synthesized by soft-templating lignin nano- and microbeads bound with cellulose nanofibrils (CNFs). The interparticle connectivity and nanoscale network in the SPs are studied after oxidative thermostabilization of the lignin/CNF constructs. The carbon SPs are formed by controlled sintering during carbonization and develop high mechanical strength (58 N·mm^–3^) and surface area (1152 m^2^·g^–1^). Given their features, the carbon SPs offer hierarchical access to adsorption sites that are well suited for CO_2_ capture (77 mg CO_2_·g^–1^), while presenting a relatively low pressure drop (∼33 kPa·m^–1^ calculated for a packed fixed-bed column). The introduced lignin-derived SPs address the limitations associated with mass transport (diffusion of adsorbates within channels) and kinetics of systems that are otherwise based on nanoparticles. Moreover, the carbon SPs do not require doping with heteroatoms (as tested for N) for effective CO_2_ uptake (at 1 bar CO_2_ and 40 °C) and are suitable for regeneration, following multiple adsorption/desorption cycles. Overall, we demonstrate porous SP carbon systems of low cost (precursor, fabrication, and processing) and superior activity (gas sorption and capture).

Anthropogenic CO_2_ emissions are believed to be a primary factor in climate change.^1^ The capture of CO_2_ from stationary emitting sites, such as power plants, is sought after as an efficient strategy to mitigate related effects.^2,3^ Conventional amine scrubbing systems are highly efficient for this purpose; however, their wide utilization is notoriously limited by the high energy consumption for operation and the corrosion of associated equipment.^4,5^ CO_2_ adsorption by solid adsorbents,^6^ such as the zeolites^7^ and metal–organic frameworks,^8^ has been demonstrated. However, porous carbonaceous materials remain as superior CO_2_ adsorbents given their stability (chemical, mechanical, and thermal) and energy intake during regeneration.^9^ Nevertheless, the diffusion of CO_2_ within carbon channels limits rapid adsorption or desorption of CO_2_, which is a factor that can be addressed by the combination of carbon nano- and microspheres that are expected to facilitate molecular transport throughout the pore network.^10^

Biobased sources, such as plant biomass, are highly attractive as carbon precursors that can be sustainably produced at large scale. As such, the preparation of bioderived carbon nano- and microspheres has been studied through hydrothermal carbonization of glucose,^11,12^ sucrose,^13^ and cellulose.^14^ In related efforts, lignin is anticipated as a convenient source, given its high carbon yield. Unfortunately, (solvo)hydrothermal carbonization of lignin typically yields interconnected, irregular topologies that lead to limitations that are similar to those reported above for typical bulk carbons.^15−17^ Herein we hypothesize that such a challenge can be resolved if one considers solid particles formed from lignin precursors that enable control over size, morphology, and composition.^18^ For such purposes, aerosol flow^19,20^ and solvent shifting^21,22^ have been demonstrated for their scalability^22−24^ and cost-effectiveness.^23,25^ Compared with glucan-based colloids, lignin particles (also referred to as colloidal lignin, lignin micro- and nanoparticles, spherical lignin, or lignin beads) have a higher carbon atom content (*ca*. 60 wt %), representing an untapped potential to create carbon nano- and microspheres. Unfortunately, recent efforts indicate that direct carbonization of lignin particles leads to fused carbon particulate aggregates, with disordered morphologies. This is explained by the partial thermoplastic behavior of lignin (relatively low glass transition temperature).^26−28^ To overcome this latter issue, preoxidation prior to carbonization has been shown to be an efficient pretreatment step.^29,30^

While lignin-based carbon nano- and microparticles are highly attractive for gas adsorption, their mobility in air or complex fluids prevents implementation as self-supported, solid and dry systems, for example, in packed columns. Therefore, the preparation of high surface area, macroscaled (yet nanostructured) carbon materials is highly desirable. Some promise pointing to this possibility is found in templated lightweight materials such as aerogels and foams,^7,31−34^ which are easily synthesized on large size scales (greater than centimeter).^32,34^ Unfortunately, these systems are usually weak and brittle, preventing deployment in adsorption systems.^35^ Loading of mineral particles (clays or silicates) has been considered as a possibility to improve the robustness of aerogels^32^ and foams;^7^ however, if used for carbon capture, such a solution defeats the intended purpose, given that mineral mining is a great contributor to CO_2_ emissions. Another alternative to enhance the mechanical strength (and to gain shape control) of lightweight materials is ice-templating; unfortunately, such an option is notorious for its energy demand.^7,32−34^

Considering the adoption of lignin for carbon capture and as a solution to the challenges presented above, we propose a supraparticle assembly process to obtain macroscale materials that simultaneously display high surface area and reactivity. Here, supraparticles (SPs) are produced with well-controlled morphology and internal networks together with a high mechanical strength.^36,37^ For this purpose, lignin particles (LPs) are combined with cellulose nanofibrils (CNFs) following evaporation induced self-assembly (EISA) to yield SPs with diameters at or above the millimeter scale. Cellulose nanofibrils form networks that induce a high interparticle adhesion and act as universal particle binder.^37^ In addition, CNFs are proposed not only to regulate the interparticle interactions during carbonization but to define the topology of the composite materials, for example, the supraparticles (SPs).

Superstructuring of nanoparticles into larger objects (SPs), as considered here for carbon capture in packed columns, facilitates handling, regeneration, and recovery, while reducing the hazards of high particle mobility, which is otherwise associated with dispersed nano- and microparticles.^36^ Hierarchical pore networks within the SPs facilitate molecular diffusion or gas transport during adsorption and desorption, which is highly beneficial in designing efficient solid adsorbents for CO_2_ capture. In this study, we thoroughly investigate the processing parameters (oxidation pretreatments and physical activation) to achieve precise control over particle network, surface area, and CO_2_ uptake capacity, as well as the mechanical integrity of the system. We demonstrate lignin-based carbon SPs with hierarchical pores spanning various length scales by the selection of the size of the lignin particles and processing conditions (Figure 1). We also numerically demonstrate the benefits of SPs when used in packed beds based on the calculated pressure drop as a function of particle size. Lastly, we systematically evaluate the influence of heteroatoms (nitrogen) on CO_2_ uptake by the carbon SPs.

**Figure 1:**
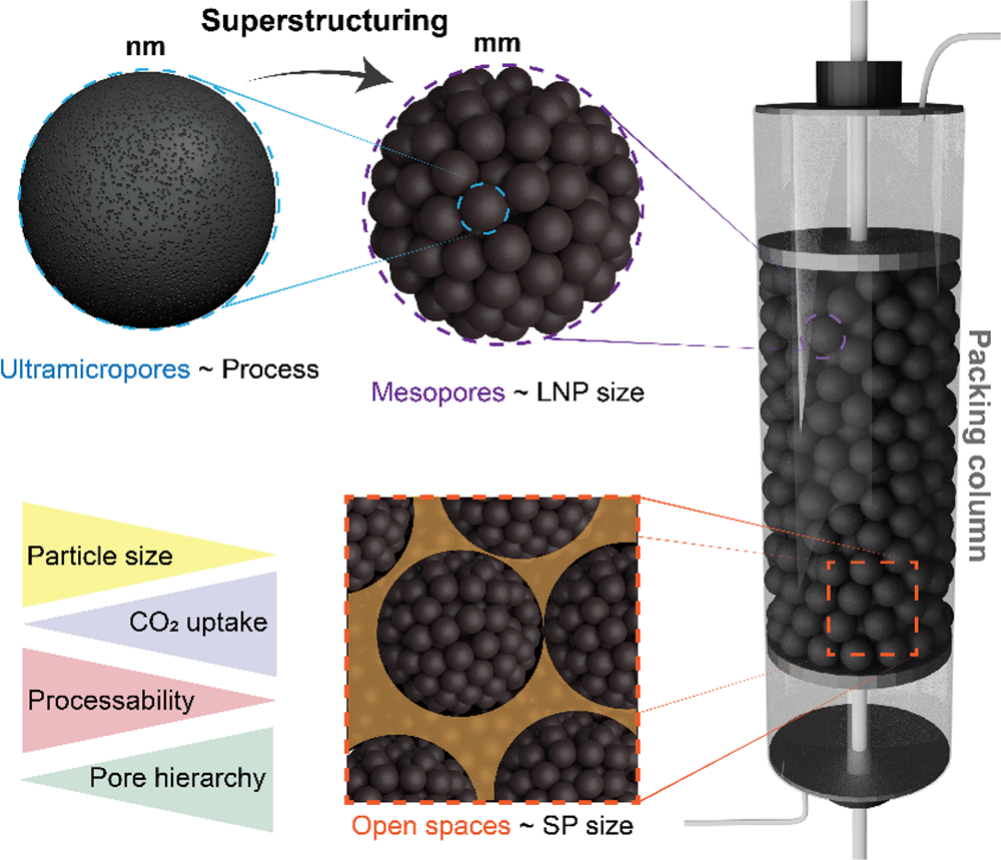
Superstructuring of lignin particles (nanometer-scale) into millimeter-scaled carbon supraparticles (SPs) for use in CO_2_ capture. The self-assembled lignin SPs are converted into carbon-based, hierarchically structured materials following controlled oxidation and carbonization. Microporosity is introduced by selecting appropriate conditions, that is, physical activation, while mesoporosity emerges from the nanoparticles, whose sizes allow the formation of mesoscaled interstitial spaces. The supraparticle packing in a bed or column allows for large void volume that facilitates gas transport while minimizing the pressure drop.

## Results and Discussion

### Oxidative Thermostabilization of Lignin Particles (LPs)

**Figure 2:**
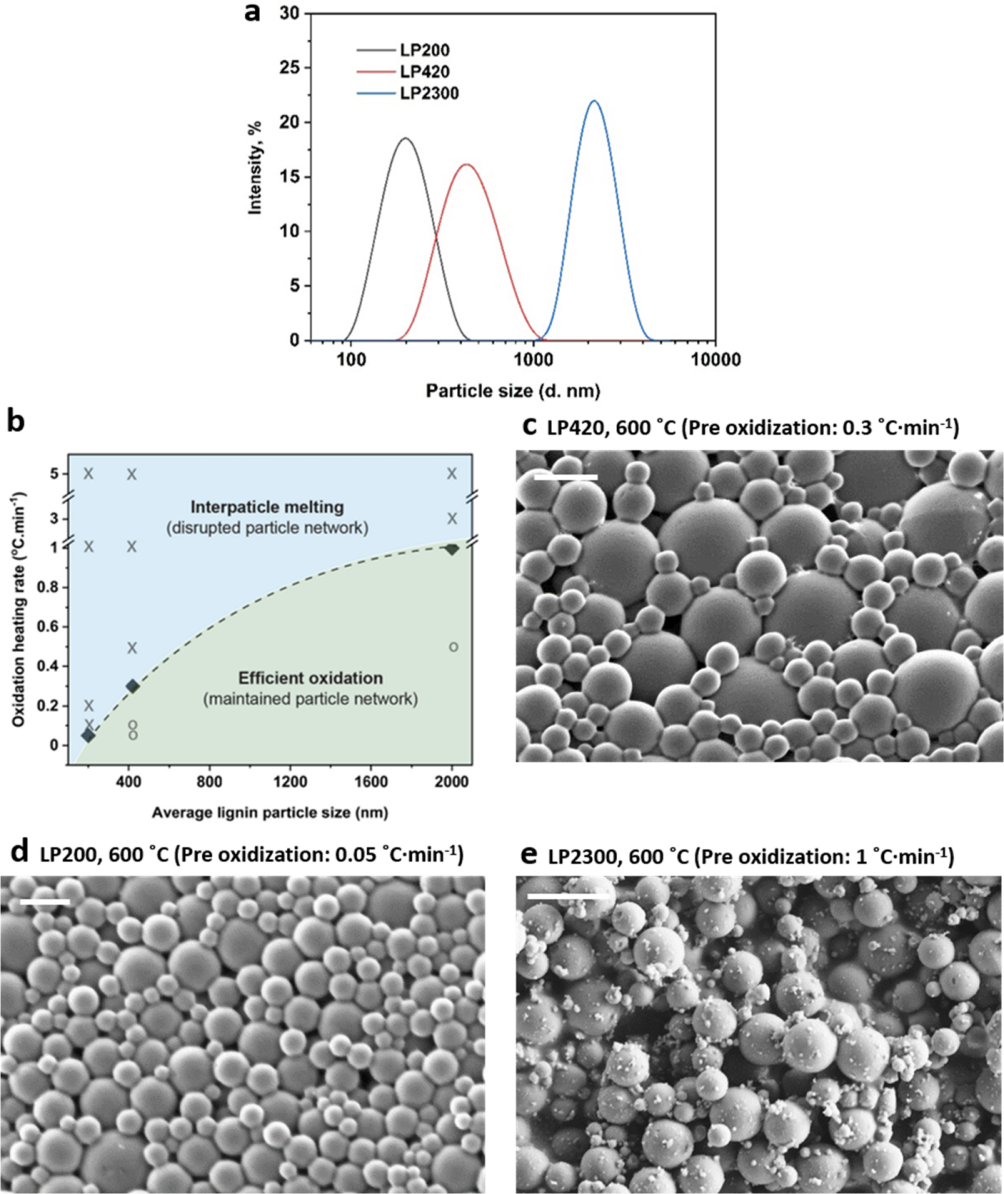
(a) Size distribution of lignin particles LP200, LP420, and LP2300. (b) Bidimensional map defining a preoxidization heating rate threshold according to the LP average particle size: heating rates above the threshold value induce particle melting (marked by crosses), while the particles preserve their morphology at lower heating rates (marked by cycles and squares). (c) SEM image of carbonized LP420 subjected to preoxidation at 0.3 °C·min^–1^. Included are also the images of (d) LP200 carbonized at a preoxidization heating rate of 0.05 °C·min^–1^ and (e) LP2300 carbonized at a preoxidization heating rate of 1 °C·min^–1^. The scale bars in panels c–e correspond to 500 nm, 200 nm, and 10 μm, respectively.

We investigated the oxidative thermostabilization of three batches of lignin particles with distinctive average diameters and polydisperse size distribution, namely, LP200, LP420 and LP2300 (Figure 2a). The LP420 particles (200–800 nm with 420 nm average diameter, Figure 2a) were spherical and presented smooth surfaces (Figure S1a). After direct carbonization at 600 °C, the particles fused completely, losing their individual features (Figure S1b). To suppress such effect, we applied oxidative thermostabilization prior to carbonization (Figures S1c–f). However, such a process is energy-intensive and generally consumes high (thermal) energy (Figure S2). Therefore, the heating rate was adjusted according to the particle size following preliminary tests at various heating rates to obtain well-defined particle networks without interparticle fusion (Figure 2c). The heating oxidation ramp used was systematically investigated for LPs (diameter from 200 nm to 2.3 μm, Figure 2b). For example, a preoxidation heating rate of 0.3 °C·min^–1^ was found to preserve the individual LP420 particles and, simultaneously, created a well-organized interparticle network (Figure 2c).

Oxidation at low heating rates, for example, at 0.1 °C·min^–1^, yielded smooth LP420 particles with excellent integrity (Figure S1f). Reactions, such as dehydration, condensation, and cross-linking involving the formation of esters and anhydrides and elimination reactions, were expected to occur during oxidative thermostabilization, increasing the glass-transition temperature (*T*_g_).^26,38,39^ At low heating rates, the *T*_g_ increased faster than the thermostabilization temperature, reaching values close to lignin’s decomposition temperature (*T*_d_);^38,39^ under such conditions, instead of melting, the lignin directly decomposed and yielded recalcitrant carbon. This is rationalized by the fact that lignin is converted from a fusible thermoplastic into an infusible thermoset, enabling the LPs to maintain their original spherical shape upon carbonization.^39^

We note that carbon microspheres obtained from LPs without thermostabilization were distinctively dense and defective.^17,40,41^ In contrast, when subjected to oxidative thermostabilization at a heating rate of 0.05 °C·min^–1^, the 200 nm lignin particles (LP200, with sizes between 60 and 400 nm) generated nonfused carbon spheres (Figure 2d). For LP420, a heating rate of 0.3 °C·min^–1^ in the preoxidization was needed for the same purpose. Likewise, the largest particles (2.3 μm, LP2300) were ideally processed at shorter thermostabilization times (1 °C·min^–1^ heating rate, Figure 2e). Overall, a close relationship exists between the rate of preoxidation and the particle size to maintain the original shape of the LPs. Such observation agrees with the fact that the melting temperature scales with nanoparticle size.^42,43^ The smaller LPs (*e.g.*, LP200) were more prone to melting and demanded longer oxidative thermostabilization and, consequently, needed a higher thermal energy. On the other hand, for supraparticle assembly, weaker interactions existed between the larger LPs (*e.g.*, LP2300) and CNFs.^37^ Taking these facts into account, LP420 was used in the assembly of lignin-based SPs and subsequent synthesis of carbon SPs.

### Synthesis of Lignin Supraparticles

**Figure 3:**
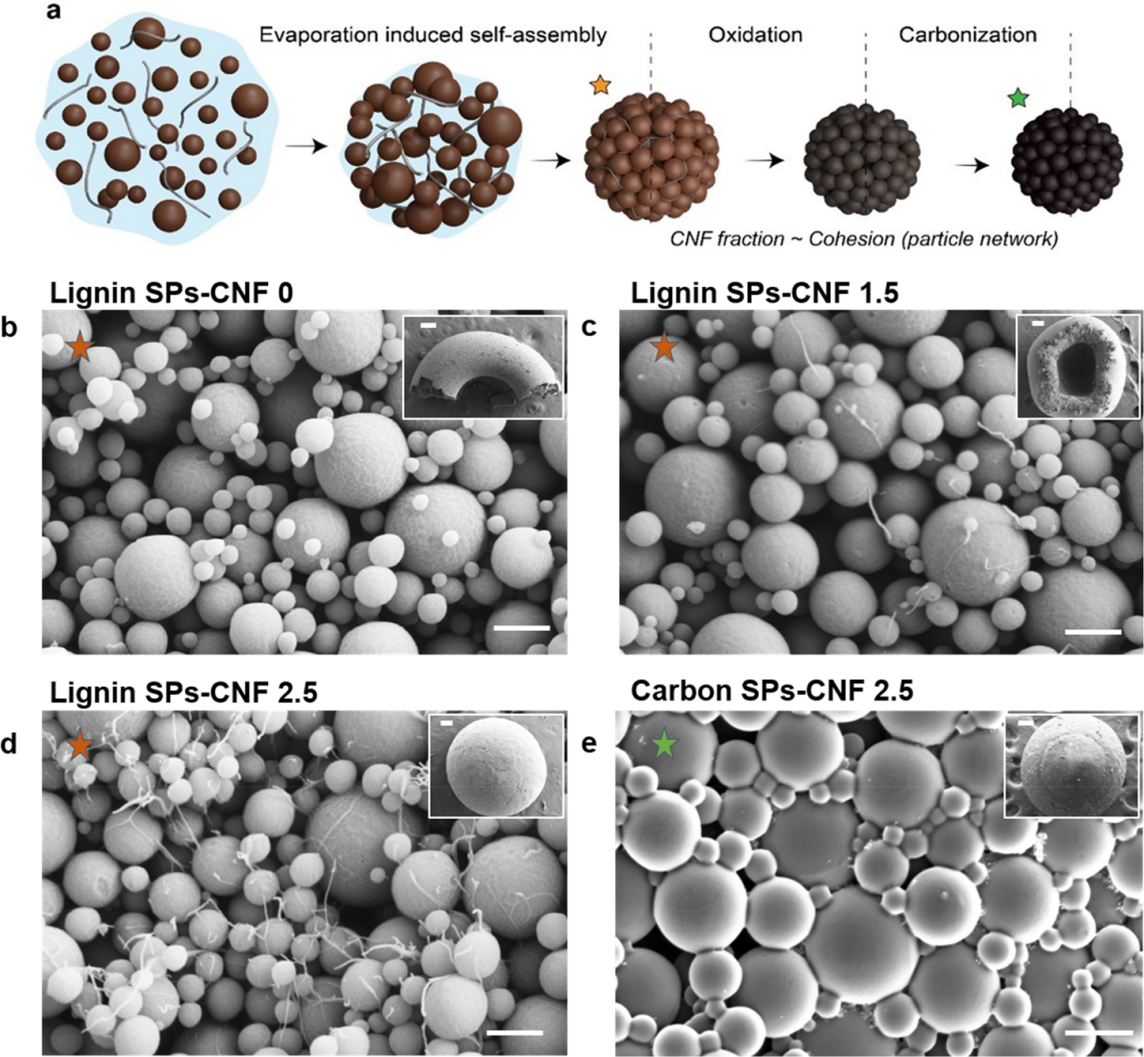
(a) Schematic illustration of the preparation of lignin SPs *via* EISA of suspension containing LPs and CNF. The process includes oxidization and carbonization to obtain carbon SPs. (b–d) SEM images showing the morphology of LP-based SPs (the insets correspond to individual SPs viewed at lower magnification). The SPs assembled from LP420 and CNF are shown at CNF mass fractions of (b) 0, (c) 1.5, and (d) 2.5 wt %. (e) Surface morphology of carbon SPs assembled from LP420 and 2.5 wt % CNF. The inset in panel e shows the corresponding carbon SPs. The scale bars in panels b–e are 500 nm, while those in the insets shown in panels b–e correspond to 200 μm.

Supraparticles of sizes in the millimeter-scale were obtained from LP420, which were assembled in the presence of CNFs that acted as interparticle binder. The SPs were prepared by evaporation-induced self-assembly (EISA), *i.e.* upon the removal of water from the bicomponent (LP420 and CNF) suspensions that were cast forming drops on superhydrophobic surfaces (Figure 3a).^36^ Supramolecular H-bonding interactions are expected to initially drive the assembly of the LPs with CNFs followed by short-range nonspecific interactions when in contact.^37^ Evaporation during ∼30 min at 60 °C yielded mechanically robust SPs (1–2 mm size) and required no further treatment. As long as the gravitational force was not significant compared to surface tension, the size of the lignin SPs could be conveniently adjusted by taking advantage of a linear relationship that we observed to exist with the casting volume. Hence, large drops (>30 μL), which are subjected to deformation under the effect of gravity, resulted in nonspherical (oblate) SPs;^36^ the remaining discussion refers to SPs obtained from drops of 20 μL.

Upon drying, shrinkage occurred, and the LPs self-assembled into larger particles, which formed as particulate networks (Figure 3a). In the absence of CNFs, evaporation resulted in ring- or doughnut-like superstructures (Figure 3b, inset). However, owing to the low interparticle adhesion, the millimetric SPs readily collapsed, liberating LPs as free small fragments (Figure S3a, the ruler is in the centimeter-scale). Addition of CNF (1.5 wt %), enabled the LP-containing droplets to form loose-packed, spherical SPs (Figure 3c, inset). We hypothesize that the formation of either doughnut- or cap-like superstructures is caused by the higher water evaporation rate near the contact line of the droplets, which are pinned during drying.^44^ At higher CNF loadings, >2.5 wt %, close-packed supraparticles were obtained (Figure 3d, inset). Such SPs, ranging from 1.5 to 2 mm in size, displayed excellent integrity, and were easy to handle (Figure S3b). Under these latter conditions, isotropic water evaporation occurred and, consequently, spherical SP shapes were produced (isotropic shrinkage generated face-centered forces, leading to compaction of LPs into a close-packed solid SP). Overall, the presence of CNF dramatically improved the formation of regular, spherical SPs comprising close-packed LPs.

The cohesion in the particulate systems was facilitated by the network formed by the cellulose nanofibrils that interacted strongly with the lignin particles.^37,45^ As shown in Figure 3b–d, the cellulose nanofibrils were distributed homogeneously across the SP structure. Both CNFs and the surface of the LPs were hydrophilic and colloidal in size, resulting in stable binary suspensions that formed homogeneous particle–CNF networks upon water evaporation.^18,37^ In polydisperse LP systems, CNF preferably interacts with the smaller particles. For example, the smaller fraction of LP420 (200–500 nm, 60% of the population) enabled SP formation, given their optimal dimensional relationship to induce cohesion in particle–fiber matrices. In fact, we found that the interstitial spaces of a particle network containing spheres ranging from 200 to 500 nm underwent an optimal dimensional coassembly with the cellulose nanofibrils.^37^ Uniform LPs of identical size are expected to enhance the mechanical performance of lignin SPs. This is because the topology of the resulting network can be suitably controlled. Additionally, during EISA of polydisperse LP420, the fraction of particles with sizes greater than 500 nm assembles with CNFs into slightly weaker constructs. LPs of greater uniformity can be produced by using lignin fractionated to display a narrow molecular weight distribution.^46−48^ However, the involved process (*e.g*., fractionation) incurs higher energy and cost, which is only justified if it adds clear benefits. In addition to facilitating SPs of enhanced mechanical strength, CNFs preserve the SP integrity (in the absence of CNFs, the superstructures disintegrated into loose LPs, as shown in Figure S3e). Interestingly, negligible or no release of LPs was observed from the surface of SPs assembled with CNFs (Figure S3f). We note that dried CNFs fit the interstices between LPs and entangled with each other, which results in a nanonetwork of nonfusing particles (or LPs fully covered by nanofibrils), thus revealing their surface for adsorption. Some dried cellulose nanofibrils were adhered to the surface of the dried LPs. These two effects contribute to the overall mechanical strength of the SPs and minimize the mobility of LPs on the SP surfaces (Figure S3f). Further control over the particle nanonetwork and resulting porosity is expected by using particles of narrower size distribution (or by using fractionated LPs), which would imply, however, an added cost.

### Supraparticle Carbonization

Carbonization of prestabilized SPs (air, 0.3 °C·min^–1^) was carried out at 600 °C in N_2_ atmosphere. The CNF-loaded SPs were converted to robust carbon SPs (∼1.2 mm), while the CNF-free SPs were released to carbon powder (Figure S3c,d, the ruler is centimeter-scaled). Fracturing and cracking is commonly observed in carbonized materials,^49^ due to anisotropic shrinkage and evolution of volatile compounds (*e.g*., CO_2_, H_2_O) throughout the channels formed during carbonization. However, this was not the case for lignin SPs, which resulted in crack-free carbon SPs (Figure 3e, inset). The “particle-of-particles” structure of the SPs facilitates gas diffusion, which prevents crack-initiation during carbonization. The carbon contribution from the cellulose fibrils (CNFs) was minor given the much higher carbon yield of the dominant component, lignin (*ca*. 45%, Table S1). This is considering not only the low CNF concentration but the relatively lower carbon content of cellulose (*ca*. 44%, Table S1), which resulted in a low carbon yield upon carbonization (∼20%, Table S1)^50^ (note some few carbonized CNFs tightly attached to the surface of carbonized LPs in Figure S4).

**Figure 4:**
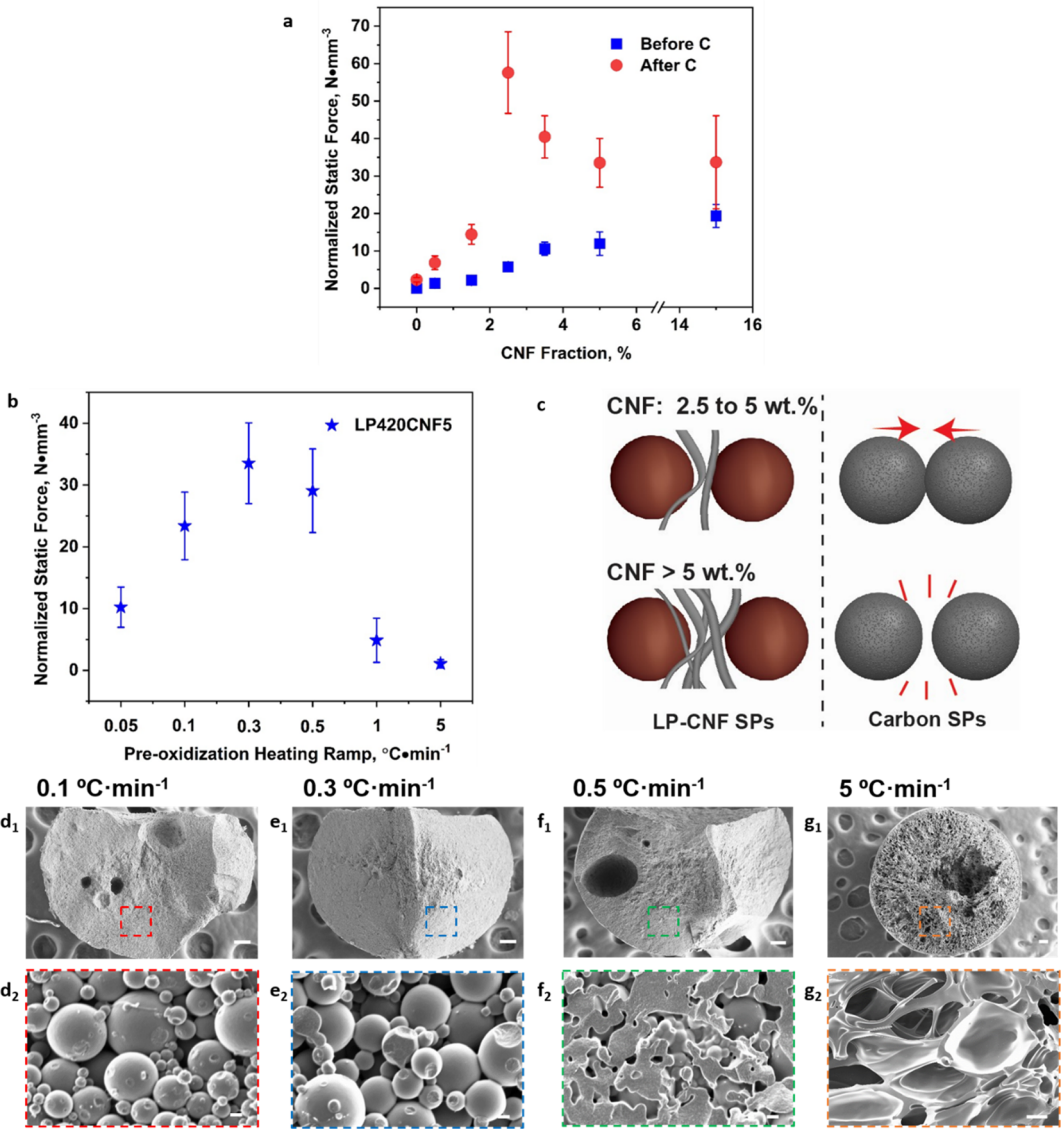
(a) Normalized (compression) static force of lignin and carbon SPs assembled by using LP420 and the given CNF fraction. (b) Normalized static force of carbon SPs assembled with LP420 and 5 wt % CNF after oxidization at the given heating rates. (c) Schematic illustration of the effect suggested for CNF in relation to interparticle networking during carbonization of LP-CNF SPs. (d–g) Crosss-section SEM images of fractured carbon SPs assembled from LP420 and 2.5 wt % CNF. The SPs were carbonized at 600 °C after oxidation in air at given heating rates. The dashed squares in panels d_1_–g_1_ show selected locations used for imaging at higher magnification, as shown in panels d_2_–g_2_. The scale bars in panels d_1_–g_1_ correspond to 100 μm, while those in panels d_2_–f_2_ are 200 nm and that in panel g_2_ corresponds to 10 μm.

Uniaxial compression tests were carried out with the lignin-based SPs to elucidate the effect of CNF content, before and after carbonization (Figure S5 and Figure 4a). In the presence of CNFs (0.5–15 wt % CNF in the final dry SPs), the supraparticles were distinctively stronger due to the adhesion introduced by the fibrils, as indicated by the measured static force at the yield point (Figure S5). The static force was normalized by the SP volume, given the nonelastic behavior and the challenges associated with attempts to fit a relation to describe the stress at failure (such as the Hertz model).^37^ Thus, the normalized static force indicated the cohesion of the SPs, which grew linearly with CNF addition, regardless of the shape of the formed lignin SPs (Figure 4a). Upon drying of the bicomponent suspension, during EISA, the cellulose nanofibrils promoted multiple physical interactions, resulting in strong interlocked 3D networks that reinforced the coassembled LP/CNF system.^37,45^

We suggest that in contrast to the LP/CNF SPs, where both interfibril and interparticle interactions are major contributors to lignin cohesion, the mechanical strength of the carbon SPs mainly depended on interparticle interactions. To test this hypothesis, we evaluated the effect of the thermostabilization step on the cohesion of the carbon SPs. The CNF content was fixed at 5 wt % while the preoxidization heating rate was varied. A clear structure–property relationship was noted (Figure 4b,d–g): we conveniently tailored the cohesion of the carbon SPs and their internal morphology by adjusting the thermostabilization conditions. After oxidation in air at a heating rate of 0.3 °C·min^–1^, the carbonized LPs partially melted inducing interconnections between neighboring LPs, which still maintained their spherical shape (Figure 4e_1_,e_2_). We speculate that under load the applied stress is distributed throughout the interconnected network, formed by the strong sub-micrometer building blocks. Thus, the carbonized SPs showed high compression resistance (Figure 4b). When the LPs were oxidized at a heating rate <0.3 °C·min^–1^, individual carbon nano- and microspheres were produced, weakening the interparticle network (Figure 4d_1_,d_2_) and consequently reducing the cohesion in the SPs (Figure 4b). If the LPs were oxidized at faster heating rates, the carbon nano- and microspheres melted and deformed, generating weak building blocks and an uneven interparticle network (Figure 4f_1_,f_2_). The latter system underwent poor stress transfer upon mechanical solicitation. Also, sharp edges of partially fused spheres acted as stress concentrators that initiated and propagated fractures. When thermostabilization was carried out at heating rates >1 °C·min^–1^, the LPs completely fused into carbon SPs with a foam-like core and a dense shell, as a consequence of gas evolution during carbonization (Figure 4g_1_,g_2_). This effect led to fragile carbon SPs (Figure 4b). Overall, the carbon SPs produced by interconnected sub-micrometer carbon spheres displayed high compression strength. Preoxidization at a heating rate of 0.3 °C·min^–1^ was used in the next efforts aimed at studying the compression strength of carbon SPs.

### Compressive Strength of Carbonized Supraparticles and Use in Packed Columns

We measured the normalized static force at the yield point of carbon SPs stabilized at a heating rate of 0.3 °C·min^–1^ and as a function of the initial CNF loading in the precursor system (Figure 4a). A high cohesion was observed and attributed to the interparticle network formed with the partially fused components interlocked by surrounding carbonized LPs. In the absence of CNF, the carbon SPs showed a marginally higher cohesion compared with the precursor lignin SPs (Figure 4a). The mechanical strength of the carbon SPs increased significantly with CNF content and reached values much higher than those of the noncarbonized (precursor) SPs. For example, at 2.5–5 wt % CNF, the carbon SPs presented a 40-fold increase in strength compared with CNF-free SPs. Meanwhile, they showed a 3-fold higher strength compared to the noncarbonized lignin SPs (Figure 4a). Marginal increases in the strength of the carbon SPs were observed at CNF loading >5 wt % (Figure 4a) (note that the cohesion of carbon SPs dramatically increased at CNF loading >2.5 wt %, which was not the case for the noncarbonized counterparts). Lignin SPs with varied CNF loadings and preoxidized at a heating rate of 0.3 °C·min^–1^ afforded the same interconnectivity during the carbonization process. The SPs experienced a structural transition as the CNF loading was increased to 2.5 wt %, from loose-packed SPs (doughnut-like or cap-like SPs) to close-packed solid SPs. In loosely packed SPs, the contribution of interconnectivity was expected to be minor. A CNF loading >2.5 wt % was critically important to compact isotropically the LPs *via* the entangled interfibril network that formed upon drying, leading to close-packed SPs. The close-packed, spherical, and isotropic carbon SPs showed the highest compression strength. The contribution of CNFs to the compression strength reached a limit once a closed-packed carbon SP was formed (Figure 4a). By contrast, excess carbonized CNFs may interfere with the interparticle interactions, which was critical to achieve compressive strength (Figure 4c). The robustness of the carbon SPs was a result of the interplay of CNF interfibril entanglement and the partially melted LPs. Lignin SPs assembled with LP420 with 2.5 wt % CNF were found to be optimal precursors of carbon SPs.

A high mechanical strength is desirable for adsorbents to be used in packed adsorption systems; for example, adsorbents in the bottom of a column should support very high or extreme loads. Thus, adsorption columns generally demand packing materials of very high mechanical stability. As such, the mechanical stability was evaluated by comparing the fracture and gravitational forces for each SP system.^44^ The gravitational force of the carbon SPs was roughly 17.7 μN. For the carbon SPs, the fracture force was over 18 N, which was about 10^6^ times larger than the gravitational force. This indicates that a close-packed bed of carbon SPs can theoretically reach a height of 1218 m before the bottom-most SPs start to break. Thus, the mechanical strength of carbon SPs is expected to fulfill or exceed the requirements of packed columns, which exclude the use of uniform LPs that would otherwise require lignin fractionation. Furthermore, nanostructured macroscaled porous materials, such as our carbon SPs, are ideally suited to minimize pressure drop in packed bed columns, such as those used for gas adsorption. According to the Ergun equation, the pressure drop in an adsorption column is highly dependent on the size of the packing particles.^51^ Here we discuss the use of supraparticles in this context, especially compared to nanosized systems.

The theoretical pressure drop along a packing column was calculated by assuming carbon spheres ranging from 420 nm to 1.2 mm and loaded in a packing bed (0.02 m diameter) (Table S2, Supporting Information). The diameter of the carbon SPs is used in the calculation regardless of the size of assembled LPs (Figure S6). The normalized pressure loss (ΔP·m^–1^) of a flue gas flowing through a column filled with carbon SPs (1.2 mm diameter) was equivalent to 33 kPa·m^–1^, Figure S7. The pressure loss (ΔP·m^–1^) rose dramatically once the particle diameter dropped to 500 μm. A normalized pressure loss (ΔP·m^–1^) determined for columns packed with carbonized LP420 was 6 orders of magnitude larger than that of the respective carbon SPs. Therefore, carbon SPs of high surface area and comprising nano- and microspheres (carbonized LP420) are clearly better suited to maintain gas exchange along a packed column, without a significant penalty in pressure drop. The “particle-of-particles” structure enables carbon SPs that can be ideally used as bulk carbon materials (Figures 4e_1_,e_2_). The macroscopic characteristics of the carbon SPs significantly improve their processability, for example, decreasing the theoretical pressure drop (ΔP·m^–1^) compared to that of carbonized LP420.

### CO_2_ Capture: Effect of Carbon SP Chemical and Structural Features

**Figure 5:**
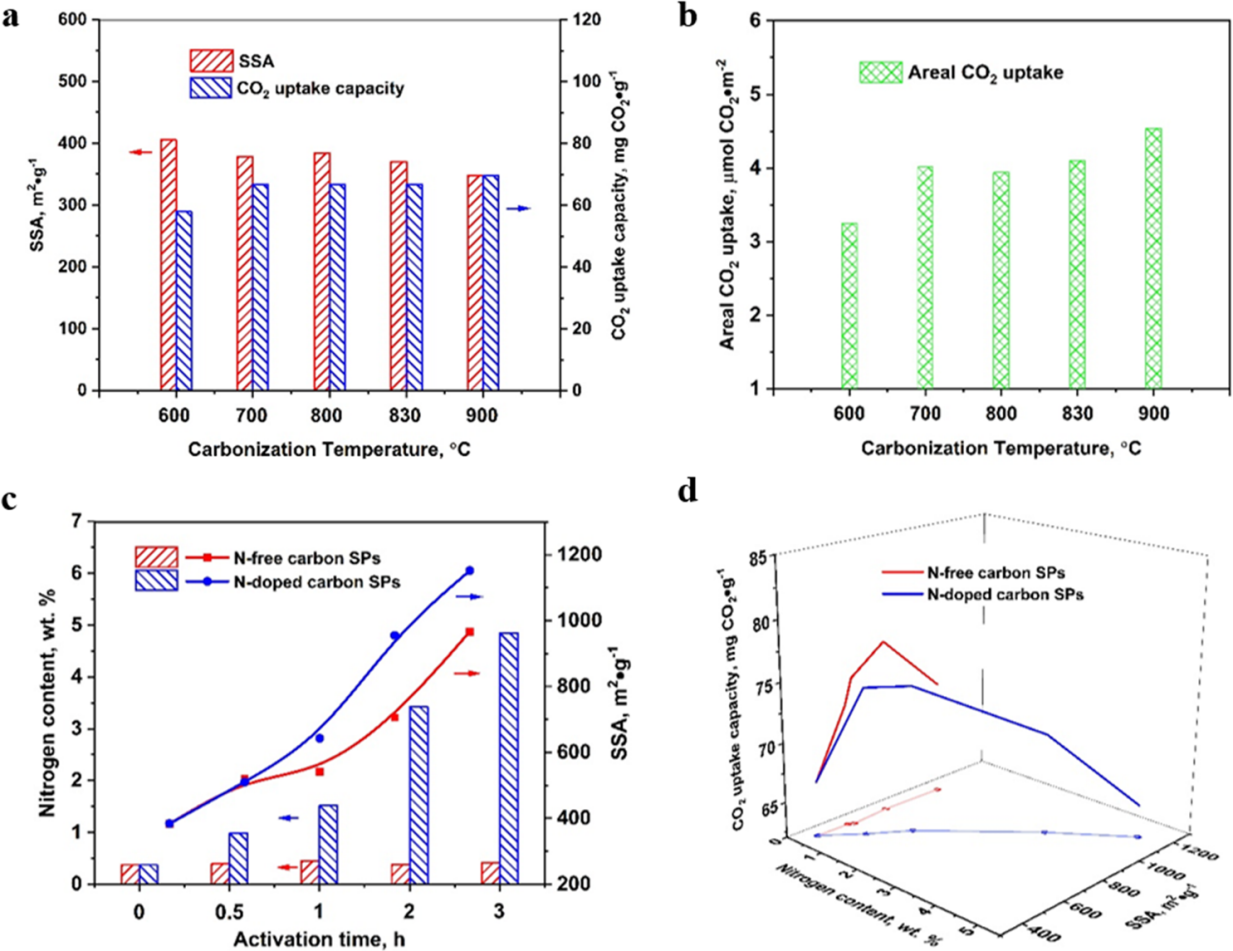
(a) Specific surface area (SSA) and corresponding CO_2_ uptake capacity as well as (b) areal CO_2_ uptake of carbon SPs obtained at varying carbonization temperatures. (c) Nitrogen content and SSA and (d) CO_2_ uptake capacity as a function of nitrogen content and SSA by carbon SPs-800 activated during given time by steam (N-free carbon SPs) and ammonia-steam (N-doped carbon SPs).

The N_2_ sorption isotherms of the carbon SPs were found to be type I with a clear peak distributed at 1 nm, characteristic of microporous structures (Figure S8a). The CO_2_ uptake capacity of the carbon SPs was measured under CO_2_ atmosphere at 1 bar and 40 °C (Figure S8b,c) and further correlated with the structural properties of the carbon SPs. If the carbonization temperature used to synthesize the SPs was raised from 600 to 900 °C, the specific surface area (SSA) of the carbon SPs slightly decreased, from 405 to 348 m^2^·g^–1^ (Figure 5a). The relatively higher SSA of the carbon SPs-600 (*i.e.*, SPs carbonized at 600 °C) led to a CO_2_ uptake capacity of 58 mg CO_2_·g^–1^. We note that the higher carbonization temperature resulted in a slightly lower SSA but, interestingly, a higher CO_2_ capture capacity. For instance, SPs-900 (SSA = 348 m^2^·g^–1^) presented a higher CO_2_ uptake capacity, 70 mg CO_2_·g^–1^. The efficiency of pores in CO_2_ uptake can be assessed by considering the areal gas capture, given in μmol CO_2_·m^–2^, Figure 5b. Interestingly, the areal CO_2_ uptake of carbon SPs was inversely related to the SSA. Thus, the SSA alone cannot explain the extent of CO_2_ adsorption. Meanwhile, it has been generally agreed that the ultramicropores (<1 nm) contribute heavily to CO_2_ uptake capacity,^52−54^ which has been confirmed to have a linear relationship with ultramicropore volume.^52,53,55^

Research efforts on the subject of CO_2_ capture have pointed to the existence of a synergistic effect of nitrogen and ultramicropores.^56−59^ In conflict with this observation, some reports have indicated that N-doping does not increase CO_2_ uptake.^54,60,61^ Meanwhile, a few comparative studies have attempted to clarify the influence of nitrogen on CO_2_ uptake in the presence of ultramicropores.^62−65^ Thus, it is still unclear whether N-doping promotes CO_2_ adsorption in carbon with ultramicropores. Therefore, we centered our next efforts on investigating the effect of N-doping on CO_2_ uptake by carbon SPs, which displayed ultramicropores. In order to avoid any ambiguities, we analyzed the effect of N-doping on CO_2_ uptake in carbon with the same structure but differing nitrogen content (Figure 5c,d). Thereafter, carbon SPs-800 were activated by (a) steam generating N-free carbons and (b) ammonia producing N-doped carbons (Figure S9).

As shown in Figure 5c, the carbon SPs-800 activated by steam became more porous, reaching up to 967 m^2^·g^–1^ after 3 h activation. The nitrogen content was identical while the pores of *ca*. 1 nm were widened slightly at high activation degree (Figure S10a). Besides introducing N heteroatoms into the carbon structure, the ammonia-steam activation producing a higher porosity within the carbon SPs-800 (Figure 5c). Both the N-content and the SSA increased with the activation time. Activation for 3 h of the carbon SPs-800 resulted in an SSA of 1152 m^2^·g^–1^ and a N content of 4.78 wt %. For the same activation time and compared to steam activation, the carbon SPs-800 presented higher SSAs when activated by ammonia-steam.

Ammonia gas has been known to produce a harsher activation compared to that of steam.^66^ Nevertheless, the pore size distribution of the carbon SPs activated by ammonia-steam was similar to that activated by steam (Figure S10a,b). Likewise, the pores of *ca*. 1 nm were also widened slightly by ammonia-steam activation. Therefore, it is reasonable to conclude that the porosity of N-doped carbon SPs-800 was similar to that of the N-free SPs at the corresponding activation time.

X-ray photoelectron spectroscopy showed pyridinic-N (N-6), pyrrolic-N (N-5), and quaternary-N (N-Q) species; the former contribute more significantly to the CO_2_ capture than N-Q counterparts,^67^ accounting for 89% of the N in the carbon SPs (Figure S11a and Table S3, Supporting Information). The nitrogen species and the corresponding fraction in the carbon SPs was consistent with previous reports^56,58^ in which the N functionality was found to enhance the affinity to acidic gas, for example, CO_2_, on carbon surfaces.

Raman spectra showed that the *I*_D_/*I*_G_ values of carbon SPs-800 increased marginally, from 1.00 to 1.02 and 1.03 after steam and ammonia-steam activation, respectively (Figure S11b, Supporting Information). This indicates that the steam activation and the introduction of N only slightly reduced the graphitization degree of the carbon SPs. The graphitization degree of the N-doped carbon was close to that of the steam activated carbon SPs-800.

So far, we have shown that the N-doped and N-free carbon SPs were nearly equal in terms of porosity, SSA, and graphitic structure but differed in their N content. Thus, a rigorous analysis of the effect of N-content on CO_2_ uptake is made possible.

Figure 5d shows the CO_2_ uptake capacity of carbon SPs-800 and the SPs activated by steam and ammonia, respectively (Figure S10c,d). The CO_2_ uptake capacity of N-free carbons first increased with a higher SSA but then dropped when the SSA reached 967 m^2^·g^–1^. The CO_2_ uptake of the N-doped carbons closely followed that of the N-free carbons (Figure 5d). The N-doped and N-free carbon SPs displayed a similar maximum CO_2_ uptake, 75 and 77 mg CO_2_·g^–1^, respectively. Thus, the incorporation of N heteroatom into the carbon SPs did not have a significant influence on CO_2_ capture. Meanwhile, the areal CO_2_ uptake of the N-doped carbons was also similar to that of the N-free carbons (Figure S11c). The carbon SPs-800, with no activation, showed the highest CO_2_ uptake, 3.94 μmol CO_2_·g^–1^, while lower values, 1.68 and 1.28 μmol CO_2_·g^–1^ were determined for carbons activated (3 h) with steam and ammonia, respectively. The CO_2_ uptake efficiency of activated carbon SPs dropped due to the gradually widened pores originally distributed at 1 nm (Figure S10a,b). The presence of N did not compensate for the “unfavorably” sized pores. The carbon SPs that presented higher ultramicroporosity showed a more extensive CO_2_ uptake, regardless of N presence.

In sum, activation can be used to tailor the porosity of the carbon SPs, for example, by the choice of activation time. However, CO_2_ capture (1 bar CO_2_ and 40 °C) with N-doped carbon SPs offered no advantage compared to N-free SPs. We note that the effect of N-doping is subject to several unaccounted variables, such as temperature (for example, CO_2_ uptake has been investigated at much lower temperatures in other efforts, 0–25 °C^56−59^).

Carbon SPs displaying a higher surface area or doped with N did not show higher CO_2_ uptake capacity. Thus, CO_2_ uptake by carbon SPs cannot be explained by the surface cover theory.^68,69^ After steam activation for 2 h, roughly 29% Kraft lignin ended up as carbon SPs, which is relatively high (Figure S12). The CO_2_ adsorption on carbon materials is typically physical (with isosteric heat between 20 and 35 kJ·mol^–1^) indicating low energy intake during regeneration.^4,56,69^

The carbon SPs can be easily regenerated (120 °C), maintaining 97–99% of the initial adsorption capacity after three adsorption–desorption cycles (Figure S13). Therefore, the introduced carbon SPs can be reused, given their physical and chemical stability. However, to confirm field applications, column systems of larger scales and subjected to longer cycles would be needed.

Finally, the reported CO_2_ uptake capacity has been shown to fall in the range between 56 and 130 mg CO_2_·g^–1^ at ∼40 °C, depending on the specific system, whether mineral, organic, or highly engineered.^70−73^ The maximum CO_2_ uptake capacity of the carbon SPs in this work (77 mg CO_2_·g^–1^) is comparable to such reported values (Table 1). At lower adsorption temperatures, the CO_2_ uptake capacity increases (48 to 168 mg CO_2_·g^–1^ at 25 °C).^4−7,65,70^ Hence, adsorption at low temperature results in a higher cooling burden for CO_2_ capture, which is energy-costly according to our previous study.^3^ Adsorption at <40 °C is not practical for CO_2_ capture, given that energy efficiency is critically important.

**Table 1:** CO_2_ Uptake or Capture Capacity of Carbon SPs Measured at 1 bar Compared to Those Reported for Other Porous CO_2_ Adsorbents

sample	adsorption temperature (°C)	CO_2_ capture capacity (mg CO_2_·g^–1^)	ref
amine-grafted solid sorbents	25	48	(6)
zeolite-loaded hybrid foams	25	53	(7)
lignocellulosic-based activated carbon	50	56	(70)
lignocellulosic-based activated carbon	25	78	(70)
microporous carbon	50	103	(71)
ordered mesoporous carbon	40	101	(72)
nitrogen-doped porous carbon	25	163	(65)
ultramicroporous carbon	25	163	(4)
hierarchical porous carbon	40	130	(73)
ultramicroporous carbon	25	168	(5)
ultramicroporous carbon	40	108	(4)
carbon SPs	**40**	**77**	this work

We note that the CO_2_ uptake performance of our carbon SPs is superior compared to that of inorganic adsorbents,^6,7^ and similar to lignocellulosic-based biochars (Table 1). Although biochars may be simple to produce, one must consider several other aspects relevant to CO_2_ adsorbent systems. For instance, compared to the typical brittle biochars, the carbon SPs are significantly more robust, given their nanostructure and are expected to support higher mechanical loads. A better control over the properties is also facilitated in the case of carbon SPs, which is advantageous for any deployment. Compared to synthetic and mineral adsorbents, the carbon SPs are more sustainable. For instance, zeolites often derive from open-pit mining, which contributes to greenhouse gas generation. Furthermore, as far as industrial feasibility, the preparation of LPs has been demonstrated to be scalable,^22,24^ cost-effective,^23,25^ and sustainable. The fabrication of lignin SPs includes simple unit operations such as mixing and casting. Typically, LP/CNF cosuspensions are dried at 60 °C, but they can be dried at ambient temperature to improve the overall energy efficiency.^37^ The current kg-level production of LPs is foreseen to open large-scale production of carbon SPs. Overall, carbon SPs obtained from inexpensive, widely available lignin show competitive performance for CO_2_ capture while presenting excellent opportunities for uses in packed bed columns and low pressure drops given the mechanical strength of the particles and their morphology and size.

## Conclusions

We synthesized robust carbon supraparticles (SPs) for applications as superior CO_2_ adsorbents. Lignin particles (LPs) with diameters ranging from 200 nm to 2.3 μm were successfully converted into nonfusible carbon nano- and microspheres by oxidative thermostabilization and subsequent carbonization. The smaller LPs were stabilized at slower heating rates, while larger lignin particles needed faster thermostabilization (LP200 can be oxidatively stabilized at a heating rate of 0.05 °C·min^–1^, while LPs of 2.3 μm required preoxidization heating rates of 1 °C·min^–1^). The mechanical strength of the assembled lignin SPs increased with increased CNF loading. Interparticle networks were generated during the carbonization when the lignin SPs were compacted by entangling nanofibrils during water evaporation *via* EISA. The synergistic effect of CNF addition and carbonization brings a significantly high level of mechanical strength to the carbon SPs, which is not reached by either of the components alone. Ammonia-steam activates the carbon SPs in a similar way compared to steam but incorporates nitrogen. These activated carbons were analyzed for the influence of N-doping on CO_2_ uptake. It was demonstrated that the nitrogen in the carbon SPs did not favor CO_2_ adsorption (1 bar CO_2_ at 40 °C). The carbon SPs can adsorb 77 mg CO_2_·g^–1^ presenting an effective and sustainable route for carbon sequestration. This work bridges microscopic and macroscopic structures based on lignin nano- and microspheres that form millimetric carbon SPs. The robust carbon SPs afforded easy handling while maintaining access to their primary surface of the carbon nano- and microspheres. These carbon SPs are expected to be superior CO_2_ adsorbents due to the better processability as bulk carbon and the rapid diffusion of adsorbates offered by the nano- and micromaterials.

## Experimental Section

### Materials

Lignin particles (LPs) with average diameter of 420 nm (herein referred to as “LP420”) were prepared following procedures described previously.^22,74^ Briefly, softwood Kraft lignin (BioPiva 100, Supporting Information) was dissolved in THF/H_2_O (3:1 mass fraction) under agitation for 3 h. The solution (4 wt % dissolved lignin) was then used to precipitate LPs under the action of deionized water that was rapidly poured in the solution, under vigorous stirring. THF was then removed by evaporation (40 °C, 30 mbar). The colloidal dispersion was purified by following several cycles of centrifugation, water removal, and redispersion in deionized water finally yielding the pure particles, without traces of THF. Particles of 200 nm average diameter were prepared following the same procedure but using a lower concentrated lignin solution (1.8 wt %).^22^ The prepared particles are herein referred to as “LP200”.

Micrometer-sized lignin particles were synthesized using an aerosol flow reactor.^19^ Briefly, pine Kraft lignin (Indulin AT, Supporting Information) was dissolved in dimethylformamide (DMF) at lignin concentration of 1 wt %. Lignin solution was atomized into droplets that were transported with a nitrogen stream under laminar flow at a given temperature. During flow-through, the solution droplets were dried into solid particles, and subsequently fractioned with a Berner-type low pressure impactor. The fraction with average particle size of 2300 nm was selected in this work, herein referred to as “LP2300”.

Cellulose nanofibrils (CNFs) were used to form superstructured assemblies of LPs. The CNFs were prepared by microfluidizing bleached sulfite hardwood (birch) fibers. The fibers were passed 6 times through a high-pressure fluidizer (Microfluidics M110P, Microfluidics Int. Co., Newton, MA) until a homogeneous gel-like aqueous suspension was obtained.

### Supraparticle Formation by Evaporation-Induced Self-Assembly

The supraparticles (SPs) were assembled by using evaporation induced self-assembly (EISA) from aqueous suspensions containing lignin particles following the procedure described by Mattos *et al*.^36,37^ In short, aqueous suspensions containing 15 wt % LPs were mixed with CNFs suspended in water (initial concentration of 1.5 wt %). Given volumes of the LP and CNF suspensions were mixed to achieve given particle-to-CNF ratios (CNF solid fraction ranging from 0.5 to 15 wt % in the final, dried SPs). The suspensions were homogenized through sequential vortex and mild (bath) ultrasonication cycles (typically three to five). Spherical SPs were assembled by casting droplets of the mixed suspension (20 μL) onto a superhydrophobic surface (Teflon-coated glass slides), followed by drying at 60 °C.

### Oxidation, Carbonization, and Activation of SPs

The dry SPs were subjected to thermal treatment (thermostabilization) at 250 °C for 2 h under a flowing air atmosphere yielding oxidized lignin-based SPs. The heating rate (from 0.01 to 5 °C·min^–1^) used in the thermostabilization step was optimized as a function of the lignin particle size. Carbonization was carried out under N_2_ flow at 600–900 °C using 1 h holding time at a heating rate of 10 °C·min^–1^. After carbonization, physical activation was achieved by using either water vapor or ammonia. The first option, steam (physical) activation, was performed by directly switching the N_2_ flow to nitrogen saturated with water vapor and keeping the flow for a given holding or activation time (for steam activation, N_2_ flow saturated with water vapor was achieved by passing nitrogen gas through a sealed bottle of Milli-Q water, as illustrated in Figure S9). At the end of the activation, the atmosphere was switched back to N_2_ until the SPs were cooled down to room temperature. Some of the obtained carbon SPs were doped with nitrogen *via* ammonia-steam activation, which was performed in an atmosphere consisting of N_2_ flow saturated with aqueous ammonia vapor at a given activation time. The same procedure used for water activation was applied except that the sealed bottle contained aqueous ammonia solution (5 wt %) instead of water. Both the Milli-Q water and aqueous ammonia solution were placed in an oil bath at 60 °C (Figure S9). All carbon SPs were washed with Milli-Q water repeatedly until neutral pH was reached, followed by rinsing with 1 M HCl and final washing with Milli-Q water. As a last step, all samples were dried in an oven at 105 °C.

### LP and SP Characterization

The diameter of the LPs was determined by using a Zetasizer Nano ZS90 instrument (Malvern Instruments Ltd., U.K.). LP suspensions were diluted with deionized water to 0.01 wt % prior to the measurement. Mean values of three replicates are reported for the particle diameter (Z-average, intensity mean).

N_2_ adsorption–desorption measurements were performed with the SPs at 77 K using a Micromeritics Tristar II equipped with an automated surface area and pore size analyzer. Prior to the measurements, the samples were degassed (Micromeritics II, Flow Prep 060) at 250 °C for 12 h under N_2_ flow. The Brunauer–Emmett–Teller (BET) model was used to determine the specific surface area (SSA), while Barrett–Joyner–Halenda (BJH) and DFT models were applied to obtain the pore size distributions.

The morphology of the SPs was observed by using field emission scanning electron microscopy (Zeiss Sigma VP, Germany) with an acceleration voltage of 5.0 kV. The samples were first coated with a 4 nm platinum/palladium layer. The carbon SPs were imaged using an acceleration voltage of 1.0 kV without any coating. The lignin and corresponding carbon SPs were fractured and imaged in the cross sections.

The elemental analysis of the carbon SPs (acclimated at 120 °C for 2 h) was carried out with an elemental analyzer (Thermo scientific, FlashSmart EA CHNS with MV) under a constant stream of helium. Carbon, hydrogen, and nitrogen content were determined in duplicate (oxygen content was determined as the balance of the other elements). Raman spectroscopy was used to identify the graphitic structure of carbon SPs. The analysis was performed using a Horiba LabRAM HR spectrometer equipped with a CCD camera and a 633 nm excitation laser.

X-ray photoelectron spectroscopy (XPS) was utilized for the surface chemical analysis of the prepared materials. The measurements were performed with an AXIS 165 (Kratos Analytical, Manchester, UK) spectrometer using a monochromated Al Kα X-ray source at 100 W. All samples were pre-evacuated overnight to stabilize ultrahigh vacuum (UHV) conditions. Both elemental wide spectra as well as high resolution regional spectra for carbon, oxygen and nitrogen were recorded.

The compression strength of the SPs was evaluated using a dynamic mechanical analysis (DMA) instrument (Q800 from TA Instruments). The compression rate was set to 4 N min^–1^, and the acquisition rate was 1 s per point.

### CO_2_ Adsorption through Gravimetry

The CO_2_ uptake by the SPs was determined using differential scanning calorimetry–thermogravimetric analyses (Netzsch STA 449 F3 Jupiter). The carbon SPs were degassed at 200 °C in He (50 mL·min^–1^, 1 bar) for 60 min and then cooled down to 40 °C under He flow. When the samples reached the adsorption temperature, He was switched to CO_2_ (50 mL·min^–1^, 99.99%) at an atmospheric pressure of 1 bar. Once the adsorption reached equilibrium at 40 °C, the amount of adsorbed CO_2_ was recorded for 60 min as a function of time. The CO_2_ uptake capacity at 40 °C was determined by the weight increase upon switching the atmosphere from He to CO_2_.

## Supporting Information Available

Lignin characteristics of Biopiva 100 and Indulin AT, SEM images of prepared lignin particles (LP420), carbonized LP420 without oxidization, and carbonized LP420 after preoxidization, flow diagram showing different oxidative heating rates, pictures of lignin-based SPs of 0 and 2.5 wt % CNF loadings, pictures of carbon SPs of 0 and 2.5 wt % CNF loadings, SEM images of LP-based SPs of 0 and 2.5 wt % CNF loadings, SEM images of carbon SPs highlighting carbonized CNF, chemical composition change and yield of pristine, oxidized, and carbonized LP420 and CNF, uniaxial compression force-strain profile of LP420-based lignin SPs prepared with 0.5–15 wt % CNF loadings, schematic illustration of the gas flow through and gas diffusion associated with packed column, the parameters of the flue gas, the theoretical pressure loss (ΔP·L^–1^) in a packing column as a function of the particle diameter (*d*_p_), pore size distribution of carbon SPs, protocol used in gravimetric CO_2_ adsorption measurements,; gravimetric CO_2_ adsorption measurements of carbon SPs, schematic illustration of activation setup, pore size distribution of carbon SPs that are activated by steam and ammonia-steam, gravimetric CO_2_ adsorption measurements of carbon SPs that are activated by steam and ammonia-steam, XPS spectra and Raman spectra of carbon SPs-800 and the SPs activated by steam and ammonia-steam after 3 h, areal CO_2_ uptake of the carbon SPs-800 activated by steam and ammonia-steam at various activation times, surface chemical composition of carbon SPs, distribution of the overall yield of steam-activated carbon SPs, and protocol used in gravimetric CO_2_ adsorption–desorption measurements (PDF)
