# Supplementary material for: Lignin-Based Porous Supraparticles for Carbon Capture
Source: ACS Nano. 2021 Mar 29;15(4):6774–86. doi: 10.1021/acsnano.0c10307 (PMC8155330; doi:10.1021/acsnano.0c10307)
Supplement: Supplementary file 1 — nn0c10307_si_001.pdf [file nn0c10307_si_001.pdf]

Supporting Information

Lignin-Based Porous Supraparticles for Carbon Capture

Bin Zhao[1], Maryam Borghei[1], Tao Zou[1], Ling Wang[1], Leena-Sisko Johansson[1], Johanna Majoinen[1], Mika H. Sipponen[2], Monika Österberg[1], Bruno D. Mattos[1,], Orlando J. Rojas[1,3,*]*

[1] Department of Bioproducts and Biosystems, School of Chemical Engineering, Aalto University, P.O. Box 16300, FIN-00076 Aalto, Espoo, Finland

[2] Department of Materials and Environmental Chemistry, Stockholm University, Svante Arrhenius väg 16 C, 106 91 Stockholm, Sweden

[3] Bioproducts Institute, Departments of Chemical & Biological Engineering, Chemistry and, Wood Science, 2360 East Mall, The University of British Columbia, Vancouver, BC V6T 1Z3, Canada.

* Authors for correspondence: Orlando J. Rojas, Email: orlando.rojas@ubc.ca; Bruno D. Mattos, Email: bruno.mattos@aalto.fi

This Supporting information document (16 pages) contains 13 figures and 3 tables.

Lignins. Softwood Kraft lignin (BioPiva 100) was obtained from UPM (Finland), which was purified from black liquor using LignoBoost technology. The molecular weight of this lignin material (Mw=5250 g·mol^{-1}, Mn=1190 g·mol^{-1}, Mw/Mn=4.4) has been characterized in our previous work.[1] Pine Kraft lignin (Indulin AT) was purchased from Meadwestvaco (U.S.A.), which was precipitated from black liquor of linerboard-grade pulp. The molecular weight of this lignin material (Mw=6549 g·mol^{-1}, Mn=656 g·mol^{-1}, Mw/Mn=9.9) is found in Ref..[2]

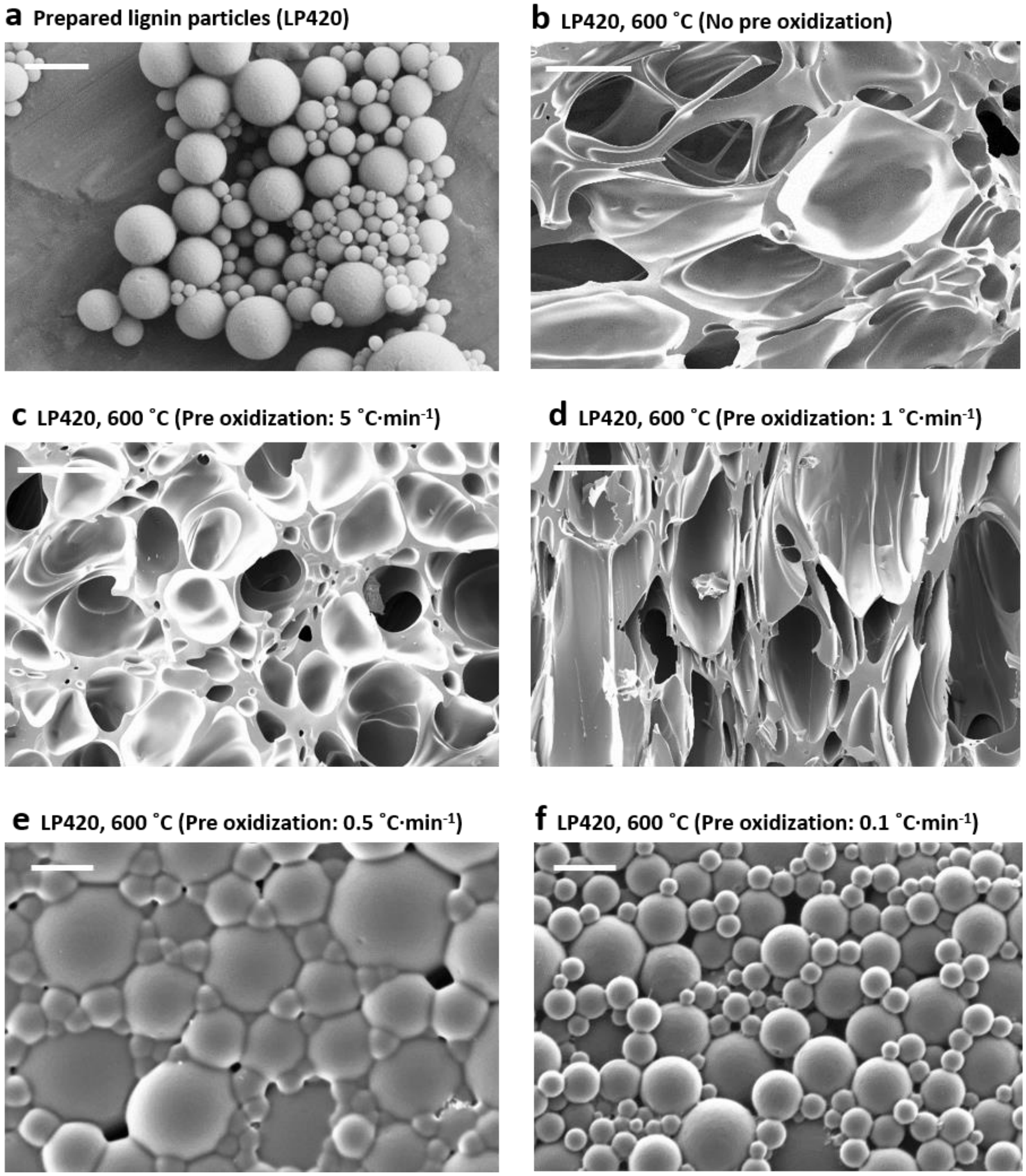

Figure S1. SEM images of (a) prepared LP420, (b) carbonized LP420 with no pre-oxidization step, and (c-f) carbonized LP420 prepared after pre-oxidization at a heating rate of 5 ºC·min^{-1} (c), 1 ºC·min^{-1} (d), 0.5 ºC·min^{-1} (e), 0.1 ºC·min^{-1} (f). The scale bars correspond to (a) 1 um, (b-d) 20 um, and (e-f) 500 nm.

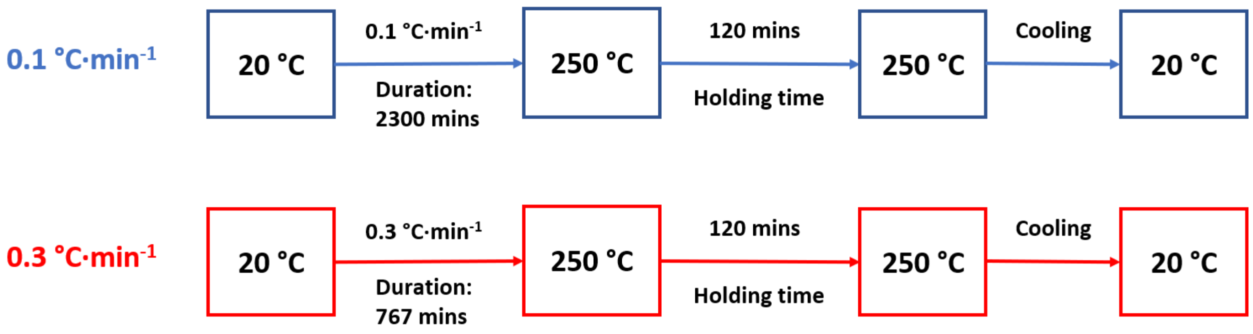

Figure S2. Flow diagram of pre-oxidization process following different oxidative heating rates. The pre-oxidization heating rate defines the duration of the oxidative thermostabilization, which used (thermal) energy and compressed air. The simplified scheme shown here indicates the duration of different steps of thermostabilization.

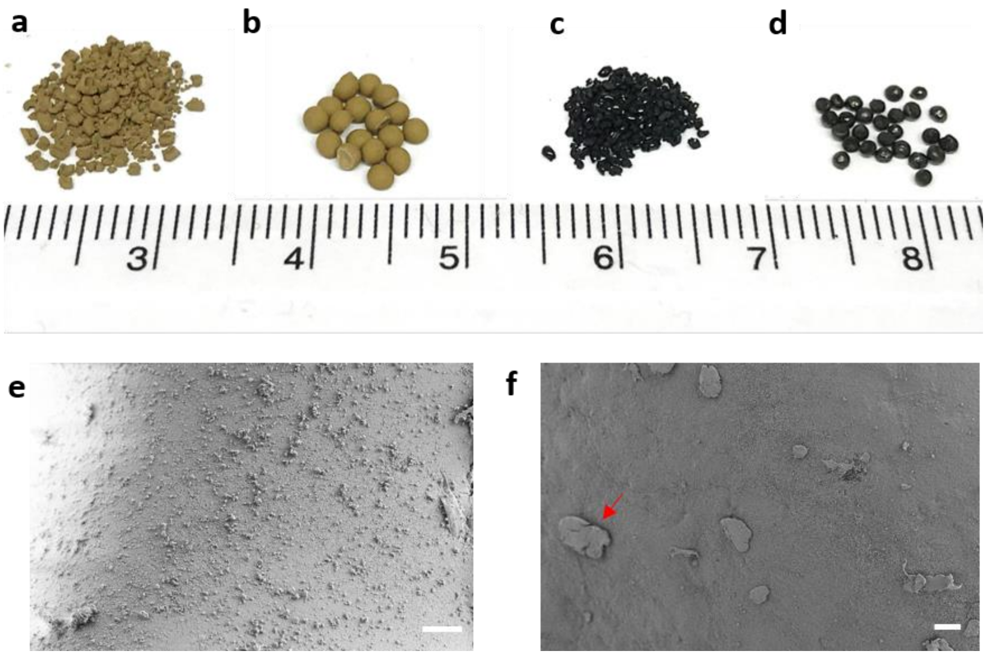

Figure S3. (a-b) Photos of lignin-based SPs: CNF-free (a), and LPs with 2.5 wt.% CNF loadings(b); (c-d) Pictures of carbon SPs: CNF-free (c), and with 2.5 wt.% CNF loading (d). The number on the ruler is shown in centimeter. SEM images showing the surface morphology of LP-based SPs: CNF-free (e), and with 2.5 wt.% CNF loadings (f). The scale bar in (e-f) is 20 μm. Note that the large particles (*e.g.* the one pointed by red arrow) on the SPs surface in (f) correspond to Teflon particles used on the hydrophobic surface.

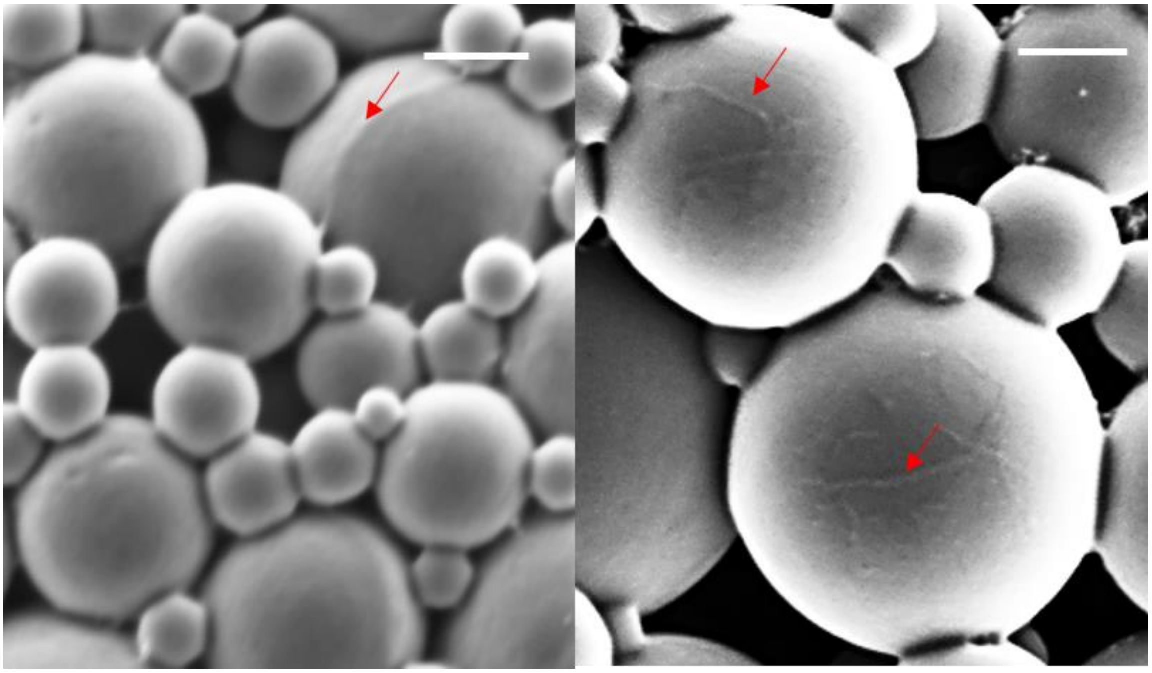

Figure S4. SEM images showing the surface morphology of carbon SPs highlighting carbonized CNF (see red arrows) on the surface of individual particles. The scale bars are 200 nm.

Table S1. Chemical composition and yield of pristine, oxidized and carbonized LP420 and CNF.

	LP420	Oxidized LP420	Carbonized LP420	CNF	Oxidized CNF	Carbonized CNF
C, wt. %	65.48	67.44	93.53	43.36	49,91	91.8
O, wt. %	26.91	25.98	6.6	50.31	38.59	7.67
H, wt. %	5.76	4.91	0.56	6.33	5.75	0.53
S, wt. %	1.85	1.67	0	0	0	0
Yield, wt. %	/	89.62	46.68	/	77.73	25.79

The chemical composition of pristine, oxidized and carbonized LP420 and CNF are shown in **Table S1**. The chemical composition of LPs and CNF throughout sample preparation (including oxidization and carbonization) are measured by Elemental Analyzer. Both the oxidization and carbonization process for the as-produced LPs and CNF are same to the conditions in which the carbon SPs are prepared. Both LPs and CNF are oxidized at 250 °C in flow air with the heating

rate of 0.3 °C·min^{-1}. Both oxidized LPs and CNF are carbonized at 800 °C in flow nitrogen with the heating rate of 10 °C·min^{-1}. After oxidization, the carbon content of LPs and CNF slightly increases while other elements, *e.g.* hydrogen, oxygen and sulfur (only for lignin), decrease slightly. After carbonization, the carbon content of LP420 and CNF increases to 93.5 wt. % and 91.8 wt. %, respectively, while other elements decrease dramatically.

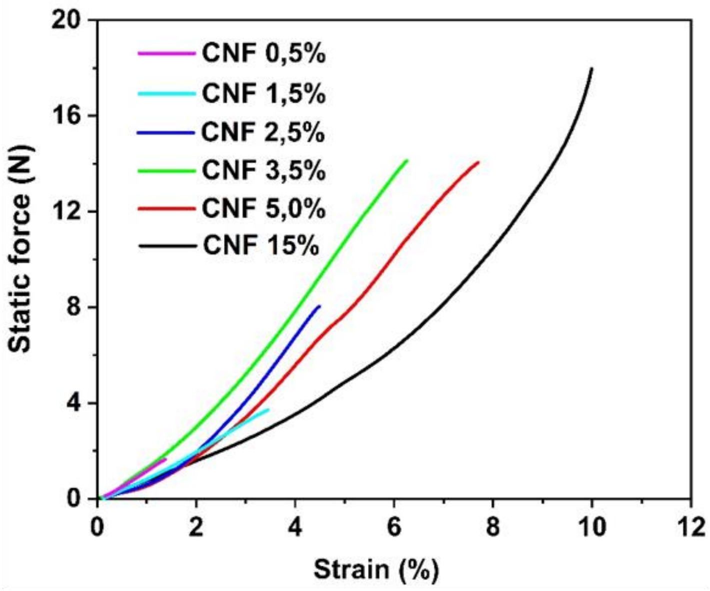

Figure S5. Uniaxial compression force-strain profile of LP420-based lignin SPs prepared with 0.5-15 wt. % CNF loadings.

In order to illustrate the dependence of theoretical pressure drop on particle size, it was assumed that carbon particles, ranging from 420 nm to 1.2 mm, were installed in a packed column with a diameter of 0.02 m (d_t). The normalized pressure loss ($\Delta P \cdot m^{-1}$) was calculated by using Ergun equation (Eq. 1).[3] The average diameter (d_p) of the particles was adopted in the calculation regardless of the polydispersity.

The bulk gas flow (dashed arrow) and the gas diffusion (solid arrow) associated with the operation of the adsorption column packed with carbon SPs are illustrated in **Figure S6**. The meso-

scaled interstitial spaces emerges from the assembly of LPs (light green spaces), which are defined by the LP size. The interstitial spaces among the packed carbon SPs (light indigo spaces) are the mm-scale, which allow the gas flow through (dashed arrow) and is factored in the pressure drop. The uniformity of the LPs influence the homogeneity of mesopore size while the gas diffusion is independent of mesopore size. We speculate that when the flue gas flows through the open voids (light indigo spaces), CO_2 molecules diffuse from the surface to the center of the carbon SPs due to the CO_2 gradient. The gas diffusion inside individual carbon SPs will not affect the pressure drop. The diameter of the carbon SPs is used in the calculation regardless of the various sizes of assembled LPs.

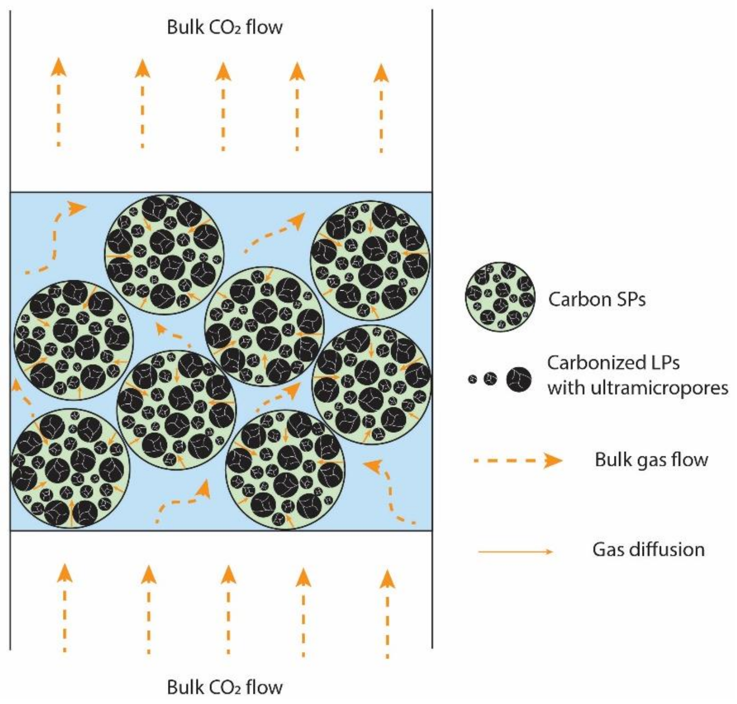

Figure S6. Schematic illustration of the gas flow through and gas diffusion associated in packed column installed by carbon SPs.

The particles were considered as spherical, namely Φ=1, since carbon SPs are round-shaped. The void fraction of the packed bed is usually between the random loose packing and random close

packing. The packing density fall between the maxmim packing density of 63.4 % and the minmum packing density of 55 %.[4] The void fraction was chosen from the ratio of the packing bed diamater to the sphere diameter (d_t/d_p ratio). An average void fraction (ε) of 38% was assumed given that the d_t/d_p ratio vary from 16.7 to several thousands in this work.[5] The condition of the flue gas is summarized in **Table S2**. The superficial velocity of the flue gas in the column is 1 $m \cdot s^{-1}$. The normalized pressure loss ($\Delta P \cdot m^{-1}$) as a function of the particle diameter are shown in **Figure S7**.

Table S2. The parameter of flue gas.

	Pressure, kPa	Temperature, ˚C	Volumetric flow, 10^{-4} $m^3 \cdot s^{-1}$	Density, $kg \cdot m^{-3}$	Viscosity, 10^{-5} Pa.s
Flue gas	110	40	3.14	1.22	1.8

$$\frac{\Delta P}{L} = \frac{150\mu(1-\varepsilon)^2\mu_0}{\varepsilon^3(\Phi d_p)^2} + \frac{1,75(1-\varepsilon)\rho\mu_0{}^2}{\varepsilon^3(\Phi d_p)^2} \qquad (1)$$

ΔP= The pressure drop,

L=the height of the bed,

μ=the fluid viscosity,

μ_0=the fluid superficial velocity,

d_t=the diameter of a packed bed,

d_p=the particle diameter,

ρ=the density of the fluid,

ε=the void space of the bed,

Φ=sphericity of the particle,

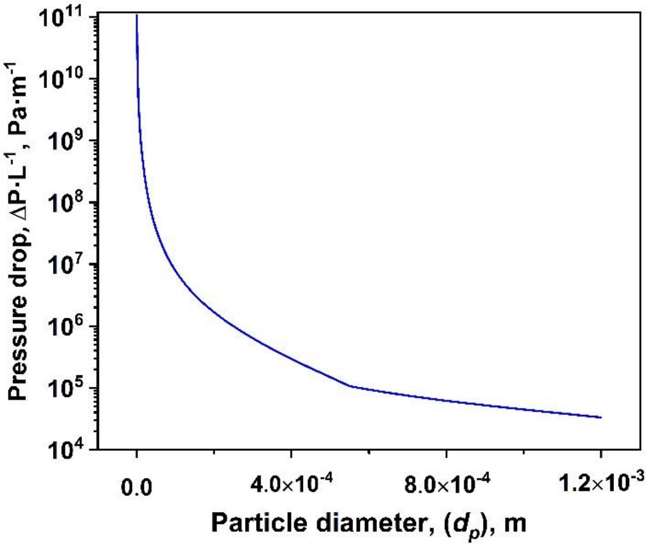

Figure S7. The theoretical pressure loss ($\Delta P \cdot L^{-1}$) in a packing column as a function of the particle diameter (d_p).

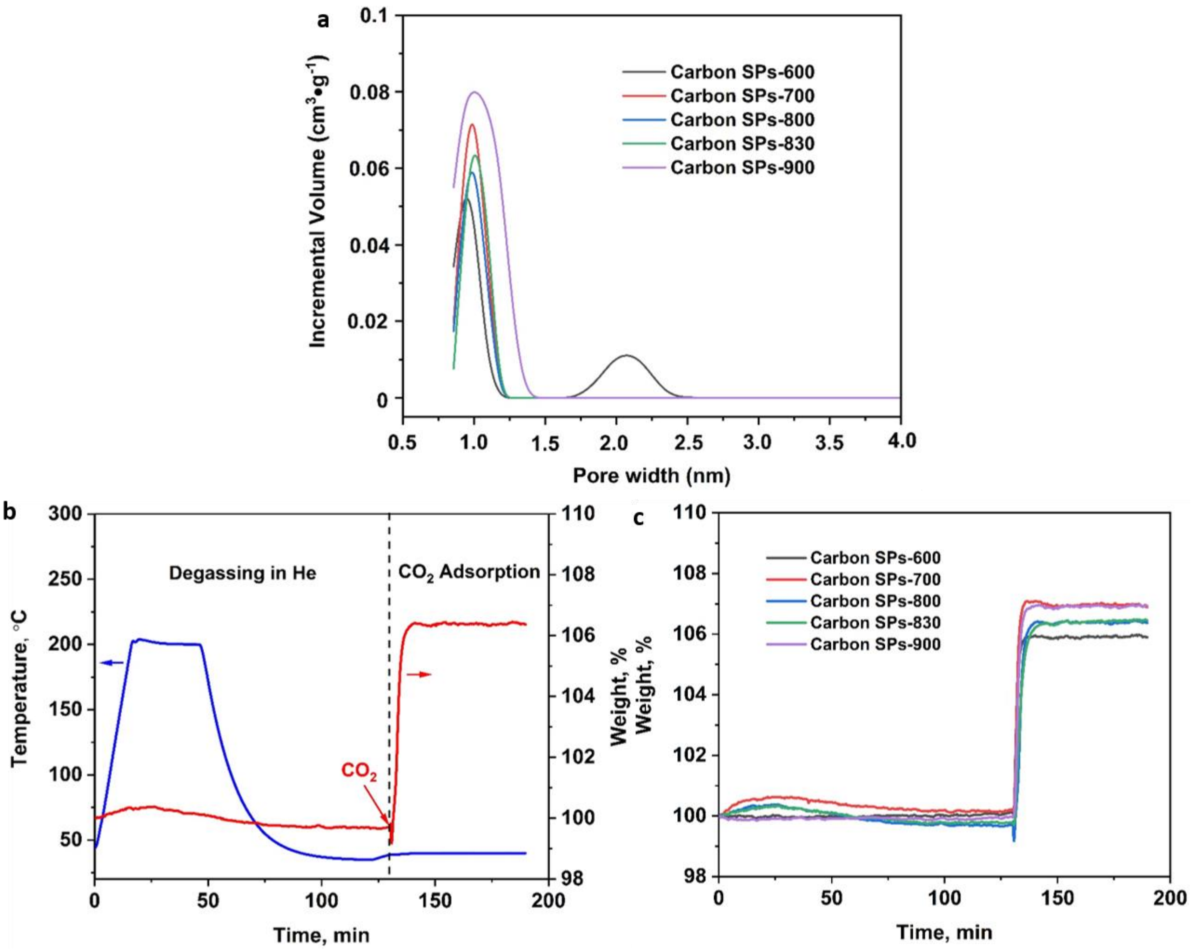

Figure S8. (a) Pore size distribution of carbon SPs-X (X represents carbonization temperature, 600-900 ˚C); (b) Protocol used in gravimetric CO_2 adsorption measurements; (c) Gravimetric CO_2 adsorption measurements of carbon SPs-X.

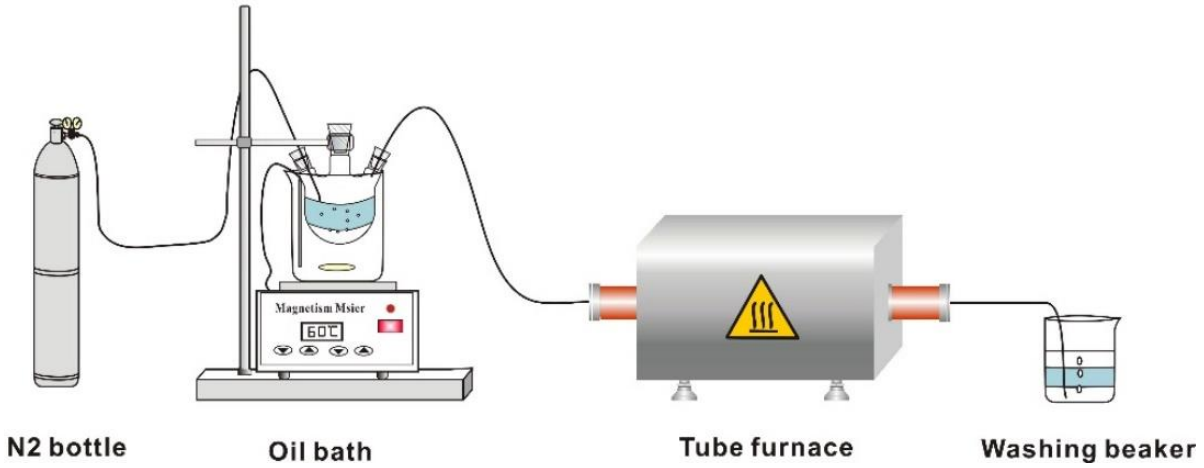

Figure S9. Schematic illustration of steam and ammonia-steam activation.

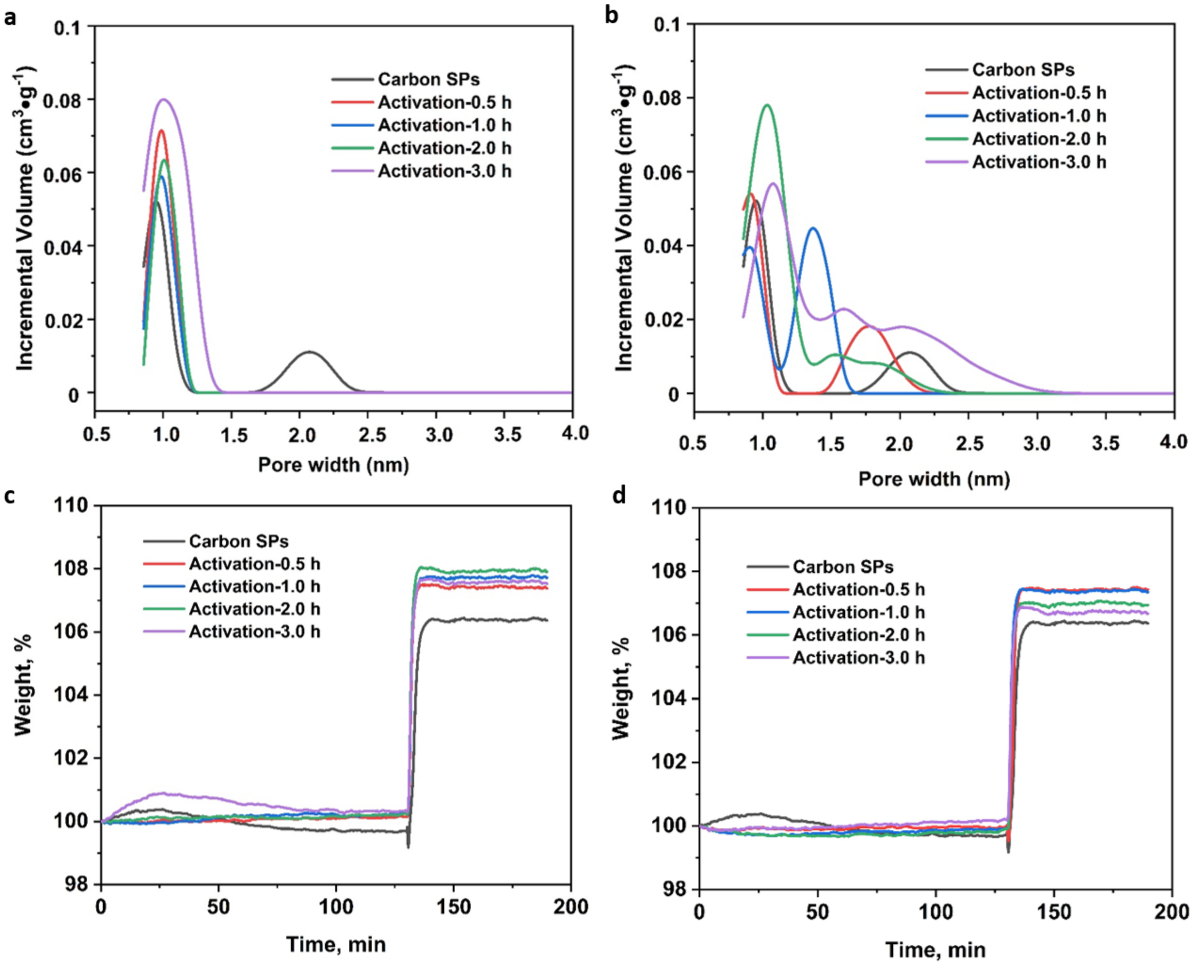

Figure S10. Pore size distribution of carbon SPs that are activated by steam (a), and ammonia-steam (b); Gravimetric CO_2 adsorption measurements of carbon SPs that are activated by steam (c), and ammonia-steam (d).

XPS spectra of carbon SPs-800 and the SPs activated by steam and ammonia-steam after 3h are shown in **Figure S11a**. The XPS spectra of all carbon samples contained only three apparent peaks assigned to C 1s (285 eV), N 1s (400 eV), and O 1s (532 eV).[6] The N 1s peaks were deconvoluted (inset of **Figure S11a**). The peaks at 398, 400 and 401.5 eV were attributed to the pyridinic-N (N-6), pyrrolic-N (N-5) and quaternary-N (N-Q), respectively.[7] As listed in **Table S3**, 4.7 wt. % of elemental N was successfully incorporated to the carbon surface, which was almost the same as the bulk nitrogen content (4.78 wt. %) obtained from elemental analysis. **Figure S11b**

shows the Raman spectra of carbon SPs-800 and the SPs activated (3h) by steam and ammonia-steam. Two featured broad bands at 1330 cm^{-1} (D band) and 1590 cm^{-1} (G band) were clearly present in all carbon samples. The D-band corresponded to the disordered graphite, while the G band reflected the ideal graphitic lattice of carbonaceous materials. The intensity ratio of D to G bands (*i.e.*, I_D/I_G) represented the degree of graphitization; that is, high I_D/I_G ratios indicated large defects in the structures.[6,8]

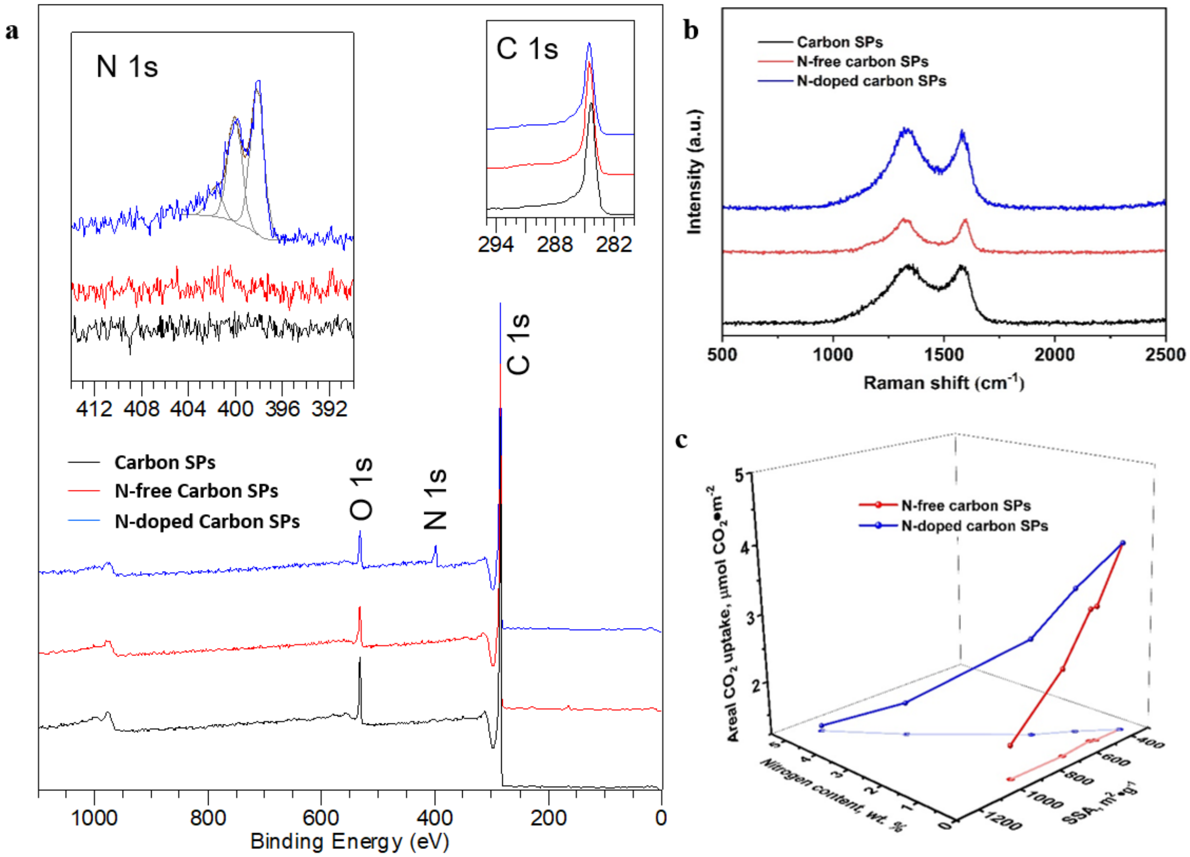

Figure S11. (a) XPS spectra (inset: C1 s spectra and deconvolution of N1 s spectra), and (b) Raman spectra of carbon SPs-800 and the SPs activated by steam and ammonia-steam after 3h; (c) Areal CO_2 uptake of the carbon SPs-800 that activated by steam and ammonia-steam, respectively at various activation time.

Figure S12. shows the distribution of the overall yield of steam-activated carbon SPs. Lignin particles (LPs) were prepared using solvent shifting, with a typical yield of 70-88%, depending on several variables, *e.g.* lignin solubility and the concentration of lignin solution. In this study, the yield of the LPs was ~ 80 %. Lignin supraparticles were fabricated by evaporation-induced self-assembly (EISA) of aqueous co-dispersion of LPs and cellulose nanofibrils (CNF). The yield of lignin SPs was as high as 98 %. The carbonization of lignin SPs can typically yield 45 % carbon SPs. The yield of steam-activated carbon SPs is defined by the activation time. After steam activation for 2 h, the yield of activated carbon SPs was around 82%. All in all, about 29 % kraft lignin was converted to activated carbon SPs. Although general lignin-derived carbon shows a slightly higher carbon yield, the carbon SPs exhibited much higher mechanical strength and hierarchical pore structures.

Table S3. Surface chemical composition of carbon SPs.

**Sample**	**C 1s (wt. %)**	**O 1s (wt. %)**	**N 1s (wt. %)**	**N-6, 398 eV (wt. %)**	**N-5, 400 eV (wt. %)**	**N-Q, 401.5 eV (wt. %)**
Carbon SPs-800	93.1	6.6	0.3		/	
N-free carbon SPs	95.6	4.2	0.3		/	
N-doped carbon SPs	90.9	4.4	4.7	51	38	11

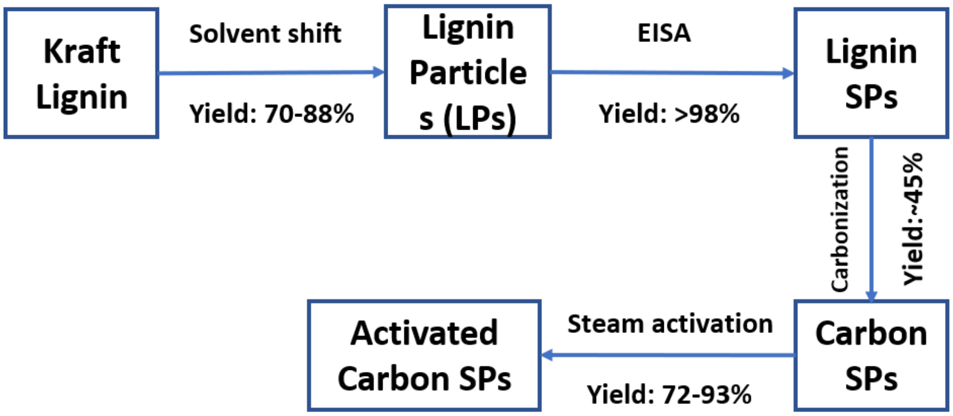

Figure S12. Distribution of the overall yield of steam-activated carbon SPs.

CO_2 adsorption-desorption measurements following three cycles are shown in **Figure S13**. Carbon SPs were degassed in N_2 at 200 °C for 120 mins. The adsorption was carried out at 40 °C under CO_2 atmosphere and then the adsorbed CO_2 was released by purging N_2 at 120 °C. In the third cycle, carbon SPs showed a CO_2 uptake capacity of 75 mg $CO_2 \cdot g^{-1}$, which is very close to the initial value, 77 mg $CO_2 \cdot g^{-1}$. In this cyclic measurement, carbon SPs maintained 97-99% of the adsorption capacity after three adsorption-desorption cycles.

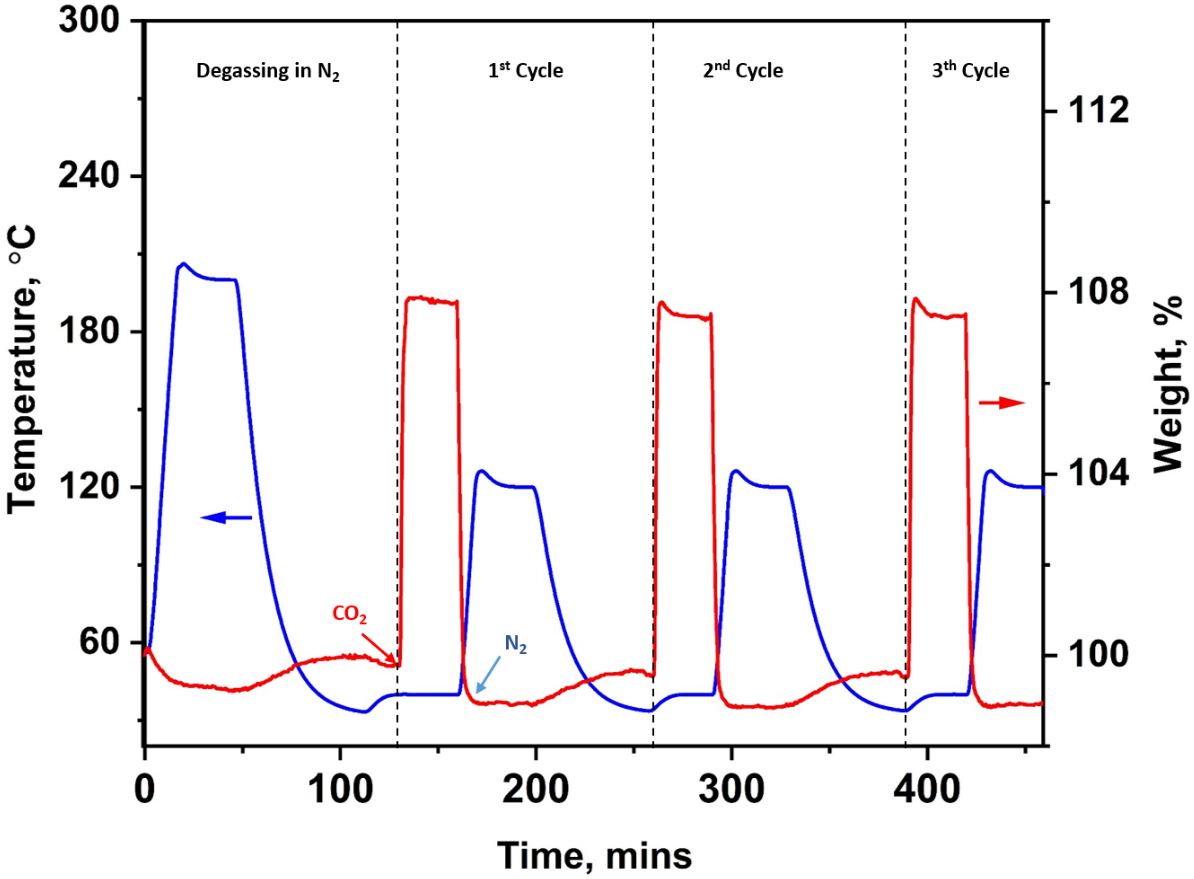

Figure S13. Protocol used in gravimetric CO_2 adsorption-desorption measurements of three cycles.

REFERENCES

(1) Sipponen, M. H.; Farooq, M.; Koivisto, J.; Pellis, A.; Seitsonen, J.; Österberg, M. Spatially Confined Lignin Nanospheres for Biocatalytic Ester Synthesis in Aqueous Media. *Nat. Commun.* **2018,** *9*, 2300.

(2) Hu, Z. J.; Du, X. Y.; Liu, J.; Chang, H.-M.; Jameel, H. Structural Characterization of Pine Kraft Lignin: BioChoice Lignin *vs* Indulin AT. *J. Wood Chem. Technol.* **2016,** *36*, 432-446.

(3) Ergun, S.; Orning, A. A. Fluid Flow through Randomly Packed Columns and Fluidized Beds. *Chem. Eng. Prog.* **1949**, *41*, 1179-1184.

(4) Song, C. M.; Wang, P.; Makse, H. A. A Phase Diagram for Jammed Matter. *Nature* **2008**, *453*, 629-632.

(5) Benenati, R. F.; Brosilow, C. B. Void Fraction Distribution in Beds of Spheres. *AIChE J.* **1962**, *8*, 359-361.

(6) Wang, Y. X.; Hu, X. D.; Hao, J.; Ma, R.; Guo, Q. J.; Gao, H. F.; Bai, H. C. Nitrogen and Oxygen Codoped Porous Carbon with Superior CO_2 Adsorption Performance: A Combined Experimental and DFT Calculation Study. *Ind. Eng. Chem. Res.* **2019,** *58,* 13390-13400.

(7) Yang, M. L.; Guo, L. P.; Hu, G. S.; Hu, X.; Xu, L. Q.; Chen, J.; Dai, W.; Fan, M. H. Highly Cost-Effective Nitrogen-Doped Porous Coconut Shell-Based CO_2 Sorbent Synthesized by Combining Ammoxidation with KOH Activation. *Environ. Sci. Technol.* **2015,** *49,* 7063-7070.

(8) Liu, Z.; Zhang, Z.; Jia, Z. J.; Zhao, L.; Zhang, T. T.; Xing, W.; Komarneni, S.; Subhan, F.; Yan, Z. F. New Strategy to Prepare Ultramicroporous Carbon by Ionic Activation for Superior CO_2 Capture. *Chem. Eng. J.* **2018,** *337*, 290-299.
